# Remodeling of the 3D chromatin architecture in the marine microalga *Nannochloropsis oceanica* during lipid accumulation

**DOI:** 10.1186/s13068-023-02378-0

**Published:** 2023-08-17

**Authors:** Tongtong Yan, Kexin Wang, Kexin Feng, Xiangchen Gao, Yinghong Jin, Hongping Wu, Wenfei Zhang, Li Wei

**Affiliations:** 1 Ministry of Education Key Laboratory for Ecology of Tropical Islands, Key Laboratory of Tropical Animal and Plant Ecology of Hainan Province, College of Life Sciences, Hainan Normal University, Haikou, 571158 China; 2 Hainan Observation and Research Station of Dongzhaigang Mangrove Wetland Ecosystem, Haikou, 571129 China

**Keywords:** Marine microalgae, *Nannochloropsis*, Chromatin 3D architecture, Histone modification, Nitrogen deprivation

## Abstract

**Background:**

Genomic three-dimensional (3D) spatial organization plays a key role in shaping gene expression and associated chromatin modification, and it is highly sensitive to environmental stress conditions. In microalgae, exposure to nitrogen stress can drive lipid accumulation, yet the associated functional alterations in the spatial organization of the microalgal genome have yet to be effectively characterized.

**Results:**

Accordingly, the present study employed RNA-seq, Hi-C, and ChIP-seq approaches to explore the relationship between 3D chromosomal architecture and gene expression during lipid accumulation in the marine microalga *Nannochloropsis oceanica* in response to nitrogen deprivation (ND). These analyses revealed that ND resulted in various changes in chromosomal organization, including A/B compartment transitions, topologically associating domain (TAD) shifts, and the disruption of short-range interactions. Significantly higher levels of gene expression were evident in A compartments and TAD boundary regions relative to B compartments and TAD interior regions, consistent with observed histone modification enrichment in these areas. ND-induced differentially expressed genes (DEGs) were notably enriched in altered TAD-associated regions and regions exhibiting differential genomic contact. These DEGs were subjected to Gene Ontology (GO) term analyses that indicated they were enriched in the ‘fatty acid metabolism’, ‘response to stress’, ‘carbon fixation’ and ‘photosynthesis’ functional categories, in line with the ND treatment conditions used to conduct this study. These data indicate that *Nannochloropsis* cells exhibit a clear association between chromatin organization and transcriptional activity under nitrogen stress conditions. Pronounced and extensive histone modifications were evident in response to ND. Observed changes in chromatin architecture were linked to shifts in histone modifications and gene expression.

**Conclusions:**

Overall, the reprogramming of many lipid metabolism-associated genes was evident under nitrogen stress conditions with respect to both histone modifications and chromosomal organization. Together these results revealed that higher-order chromatin architecture represents a new layer that can guide efforts to understand the transcriptional regulation of lipid metabolism in nitrogen-deprived microalgae.

**Supplementary Information:**

The online version contains supplementary material available at 10.1186/s13068-023-02378-0.

## Introduction

Nitrogen is an essential element required by all photosynthetic organisms [1], serving as one of the most important nutrients in both aquatic and terrestrial ecosystems wherein it shapes plant, algae, and cyanobacterial growth [2]. Plant nitrogen deficiency results in a range of physiological impairments including impaired growth, weaker photosynthetic activity, abnormal carbon partitioning, and changes in the biosynthesis of carotenoids and chlorophylls. Microalgal cells exposed to nitrogen stress often exhibit increases in carbohydrate storage in the form of polysaccharide and lipid accumulation [3]. For example, nitrogen availability has been shown to influence starch and triacylglycerol (TAG) bioaccumulation in the green microalgae *Chlamydomonas reinhardtii* [4] and *Chlorella sorokiniana* [5], the diatom species *Phaeodactylum tricornutum* [6], and red alga *Cyanidioschyzon merolae* [7]. Nitrogen deprivation (ND) has been reported to have a particularly strong enhancing effect on TAG biosynthesis in certain oleaginous microalgae including *Nannochloropsis oceanica* [8] and *Neochloris oleoabundans* [9]. ND tends to enhance TAG synthesis such that photosynthetic organisms suffering from nitrogen stress need to undergo metabolic, biosynthetic, and/or transcriptional reprogramming to adapt effectively to these stress conditions. Consistently, pronounced transcriptional reprogramming in response to ND has been documented in a range of microalgae including *C. reinhardtii*, *P. tricornutum*, *N. oleoabundans* and *Nannochloropsis* [4, 8–10]. Microalgal chromatin reorganization and histone modification reprogramming in response to ND conditions, however, remain to be fully characterized.

The three-dimensional (3D) spatial organization of the genome and associated histone modifications can have a profound impact on transcriptional activity and associated biological activities in eukaryotic cells and organisms [11–13]. Experimental approaches have been developed to effectively analyze 3D genomic architectural organization such as the Hi-C chromosome conformation capture approach, revealing that the chromatin exhibits multiple levels of local and global organization that include: (1) A and B compartments on the megabase-scale associated with respective areas of active and inactive gene expression [14]; (2) topologically associating domains (TADs) on the sub-megabase-scale that consist of basic separated 3D structural units which play important roles in the context of replication and transcriptional regulation [11]; and (3) chromatin loops that play direct roles in the regulation of transcriptional activity, including enhancer-promoter interactions. Maps of the 3D organization of the chromatin have been reported for model *Arabidopsis* [15], maize [16, 17], barley [18], tomato, Sorghum [19], and rice [20], revealing a strong association between these 3D structural characteristics, genomic functionality, and transcriptional activity. Notably, environmental changes can alter the 3D organization of the eukaryotic chromatin, as in a report in which *Arabidopsis* exposure to heat stress resulted in chromatin reorganization related to the activation of transposons [21]. Similarly, the cold treatment of rice can increase interactions between the A and B compartments while inhibiting long-range intra-chromosomal interactions, consistent with the decondensation of the whole rice genome [22]. Rice exposure to heat stress further induced A/B compartment transitions, an increase in the size of TADs, and the disruption of short-range interactions [23]. Phosphorus deficiency has further been reported to induce 3D changes in chromatin structural characteristics in *Medicago truncatula* correlated with histone modifications [24]. How chromatin organization is affected by nitrogen stress conditions, however, remains to be assessed.

*Nannochloropsis* sp. have been established as an important model organism for use in research settings as they exhibit rapid photosynthetic growth, tolerate a wide range of environmental conditions, are amenable to genetic modification, and produce high levels of TAG and eicosapentaenoic acids [25, 26]. *Nannochloropsis* sp. are particularly important tools for use in synthetic biology applications wherein they serve as photosynthetic cell factory. To date, there have been several published transcriptomic analyses of *Nannochloropsis* sp., including RNA-seq results published for *N. oceanica* CCMP1779, *N. gaditana* CCMP526, and *N. oceanica* IMET1, that offer insight into the genetic processes associated with ND-induced neutral lipid accumulation. These studies found that between 939 and 3255 genes exhibited transcriptional reprogramming in response to nitrogen stress [8, 27–29]. Whether and how 3D chromatin structural reorganization and epigenetic reprogramming at the level of histone modifications are related to nitrogen stress-related changes in transcriptional activity in *Nannochloropsis* microalgae, however, have yet to be established.

In the present study, a series of Hi-C, ChIP-seq, and RNA-seq analyses were performed to explore 3D chromatin restructuring and the relationship between chromatin characteristics and transcriptional activity in the marine microalga *Nannochloropsis oceanica* in response to ND conditions. Ultimately, these experiments revealed that nitrogen stress had an impact on A/B compartment transitions, TAD types, and long-range inter- and intra-chromosomal interactions. Integrating the results of these different analytical approaches revealed that chromatin reorganization is associated with histone modifications and shifts in patterns of gene expression. Strikingly, lipid metabolism was found to be subject to reprogramming at the chromatin structure and histone modification levels, with associated genes exhibiting more dynamic gene expression and chromatin signals, offering novel insights that may aid efforts to optimize or otherwise modulate lipid production. Together, these findings demonstrate that the 3D organization of the chromatin and histone modifications represent new regulatory layers that govern microalgal lipid accumulation-associated transcription in response to nutrient stress conditions.

## Results

### Experimental approach to analyzing *N. oceanica* under ND conditions

In an effort to explore the regulation of lipid accumulation and global metabolic activity in marine microalgae at the chromatin level, a nitrogen stress-based experimental design was employed (Additional file 1: Fig. S1A). Briefly, *Nannochloropsis* cells were initially cultured in nitrogen-replete (NR) liquid culture medium until reaching the logarithmic phase of growth (OD_750_ = 2.0; Sample C0) at which time they were transferred into media under ND conditions and cultured for either 1 day (Sample N1) or 2 days (Sample N2). The 3D chromatin structural characteristics, histone modifications, and transcriptional profiles in these three different samples (C0, N1, and N2) were then assessed over the course of lipid accumulation (Additional file 1: Fig. S1A and “Methods”). The growth and photosynthetic activity (based on photosystem II efficiency) of these microalgal cells were also analyzed under NR and ND conditions as in prior studies [30]. These analyses revealed that the growth, photosynthetic activity (*Fv/Fm*), and photosynthetic oxygen evolution (POE) of *N. oceanica* slowed by ~ 20%, ~ 18%, and ~ 25% under ND conditions as compared to NR conditions (Additional file 1: Fig. S1B). These data are consistent with previously published results [31, 32], indicating the reliability of this experimental system and the associated results.

### Analyses of ND-related changes in 3D chromatin organization

**Fig. 1:**
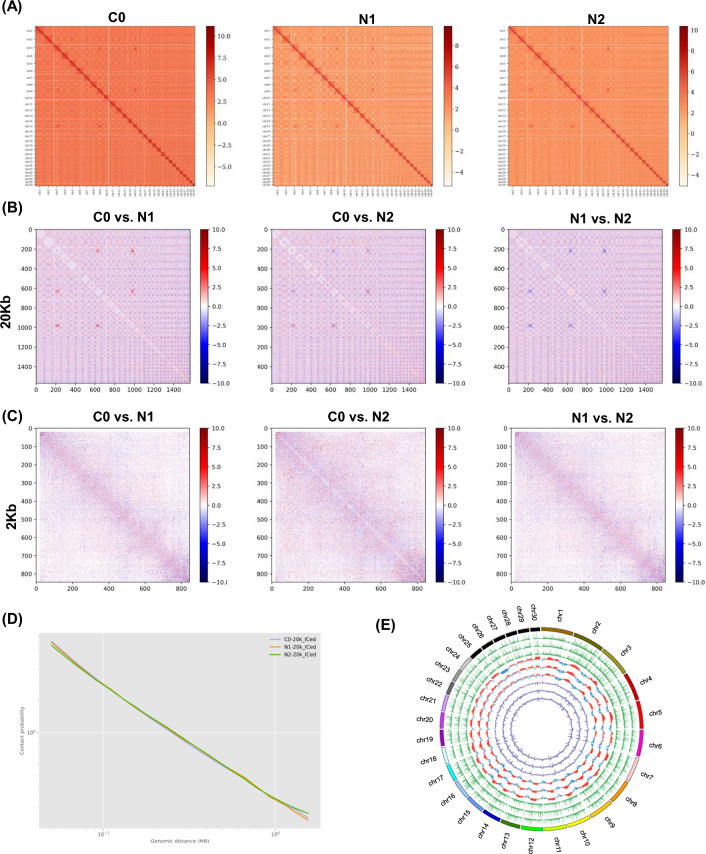
Genome-wide chromatin 3D structure dynamics in response to nitrogen deprivation in *N. oceanica*. **A** Genome-wide chromatin interaction under C0, N1 and N2 in *N. oceanica*. **B**, **C** Genome-wide difference chromatin interaction map. The 20-kb and 2-kb binned heatmap (subtractive matrix) were shown by depicting the *Z*-score difference between C0 and N1, C0 and N2, or N1 and N2 (see “Methods” for *Z*-score difference calculation). **D** Averaged scaling plot of interaction frequencies against increasing genomic distance in *N. oceanica*. The genomic bin size is 100 kb. **E** Circos plots of the chromatin 3D structure of *N. oceanica* genomes. Seven rings from the outside to inside show genomic positions, gene expression FPKM (fragments per kilobase per million mapped reads) value, PC1 signal of A compartment (red) and B compartment (blue), and TAD-separation score for C0, N1 and N2, respectively

Next, the 3D chromatin structural changes in *N. oceanica* under ND conditions were explored through a Hi-C approach. Samples from the C0, N1, and N2 time points, respectively, yielded 58.3G, 59.2G, and 65.9G of raw reads (Additional file 2: Table S1). Strong correlations (Pearson correlation coefficient > 0.94) were observed when assessing chromatin interactions in replicate samples, and 34.9, 13.1, and 24.5 million valid read pairs were ultimately generated for the respective C0, N1, and N2 samples (Additional file 2: Table S1). Valid reads produced an overall Hi-C matrix resolution of 2 kb (Fig. 1A and Additional file 1: Figure S2), using the estimation method published previously. Conformational transformations within the chromatin were examined by calculating differences between pairs of chromatin interaction maps (C0 vs. N1, C0 vs. N2) following the conversion of these data into *Z*-scores normalized for genomic distance [33]. Features consistent with results from other species were evident when binning this interaction data at intervals of 20 kb and 2 kb. Cis interactions are the most common, decaying as genomic distance increases (Fig. 1B, C). Subtraction analyses of the defined chromatin interaction map pairs highlighted clear ND-induced changes in these chromatin interactions in N1 and N2 samples relative to control C0 samples (Fig. 1B, C; Additional file 1: Figs. S3, S4). Intra-chromosomal interaction frequencies decreased rapidly as linear genomic distance increased with a primary one at 50 kb (Fig. 1D and Additional file 1: Fig. S5), although at genomic distances greater than 100 kb this frequency of interaction rose. An RNA-seq analysis was next conducted with the goal of exploring the role that chromatin topology plays in regulating transcriptional activity in these marine microalgae (Fig. 1E).

**Fig. 2:**
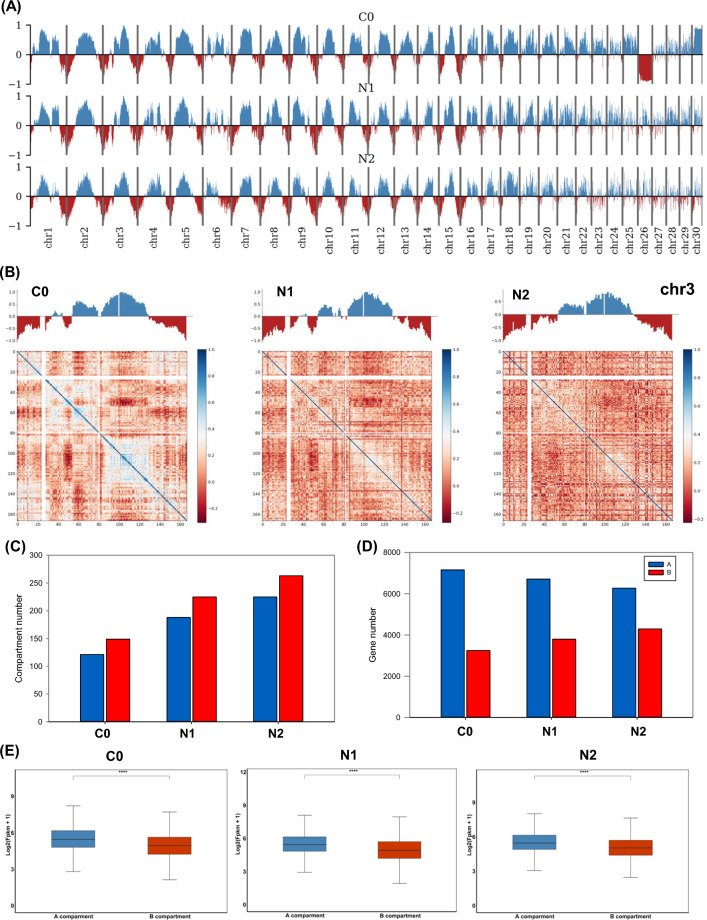
Compartment A/B analysis in response to nitrogen deprivation in *N. oceanica*. **A** Segregation of genome-wide global A/B compartments using eigenvector under C0, N1 and N2 in *N. oceanica*. Blue and dark-red represented compartment A and B, respectively. **B** Compartment A and B of chromosome 3 as an example exhibited at 10-kb resolution under C0, N1 and N2. Blue and dark-red represented compartment A and B, respectively. The upper line shows the partition of A (blue histogram) and B (red histogram) compartments. The lower track indicates the first principal component values showing A/B compartment status at 10 kb resolution. **C** The number of compartment A and B identified under C0, N1 and N2. Blue and dark-red represented compartment A and B, respectively. **D** The number of genes in the regions of compartment A and B identified under C0, N1 and N2. Blue and dark-red represented compartment A and B, respectively. **E** The gene expression level of compartment A and B analyzed under C0, N1 and N2. Blue and dark-red represented compartment A and B, respectively

A principal component analysis approach was used to divide megabase (Mb)-sized regions of the chromatin contact map into alternating positive and negative eigenvectors corresponding to the euchromatin-rich ‘A’ and heterochromatin-rich ‘B’ compartments (Fig. 2A). These two compartments comprise the secondary structural organization of the chromatin, with these distinct territorial chromatin subdomains having been documented in a wide range of plant and animal species [19]. These A and B compartments were evident throughout the *N. oceanica* genome (Fig. 2A), in line with reports from studies of mammalian and plant species [34]. These compartments were defined for each chromosome (Fig. 2B; Chromosome 3 as an example), revealing that B compartments comprised a higher overall proportion of the *N. oceanica* genome. In total, 121, 188, and 225 A compartments were delineated in the C0, N1, and N2 samples, respectively, as compared to 149, 225, and 263 B compartments (Fig. 2C). The A compartment comprised 63.11%, 57.70%, and 53.20% of the genomic regions in the respective C0, N1, and N2 samples while the B compartment comprised the remaining 35.13%, 40.63% and 45.02% (Additional file 2: Table S2). The A compartment regions in these three respective samples included 2252, 2484, and 2588 genes (Fig. 2D), with significantly higher gene density in compartment A relative to compartment B (Additional file 1: Fig. S6A), while the GC content of compartment B was significantly higher than that of compartment A (Additional file 1: Fig. S6B). Significantly higher levels of gene expression were also evident in compartment A regions as compared to compartment B regions in all three samples (Fig. 2E). Notably, high levels of A/B compartmental switching were evident in cells exposed to ND conditions, with 285, 403, and 290 A–B compartment switch regions and 118, 97, and 152 B–A compartment switch regions identified within the *N. oceanica* genome for the respective C0 vs. N1, C0 vs. N2, and N1 vs. N2 comparisons (Additional file 2: Table S3). Of the 30 chromosomes comprising the *N. oceanica* genome, the highest levels of chromatin reorganization were evident for chromosomes 23, 26, and 30 when comparing the C0 and N1 samples (Additional file 1: Fig. S7). No significant differences in gene density or GC content were observed in the regions subject to A/B compartmental switching under ND conditions (Additional file 1: Fig. S8A, B).

**Fig. 3:**
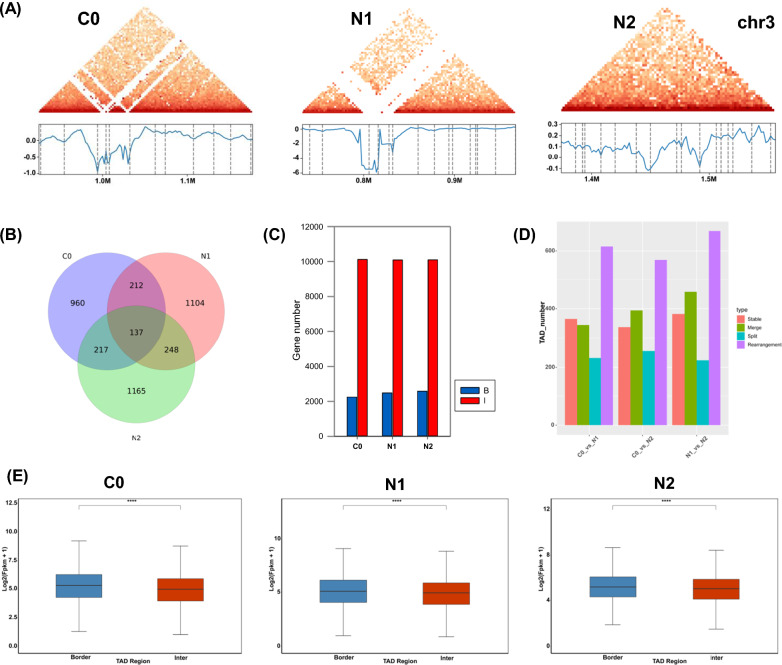
TAD analysis in response to nitrogen deprivation in *N. oceanica*. **A** TAD of chromosome 3 as an example exhibited under C0, N1 and N2. Blue and dark-red represented TAD border and inter, respectively. **B** Venn diagram of TAD under C0, N1 and N2, respectively. **C** The number of genes in the regions of TAD border and inter identified under C0, N1 and N2. Blue and dark-red represented TAD border and inter, respectively. **D** The transition of TAD in response to nitrogen deprivation under C0 vs. N1, C0 vs. N2, and N1 vs. N2. TAD transition was firstly defined as four types including ‘Stable’ (the overlapped proportion of the TAD in two treatments was above 0.75), ‘Split’ (one TAD divided into two or more TADs), ‘Merge’ (two or more TADs fused into one TAD), and ‘Rearrangement’ (remaining situations) [36]. **E** The gene expression level of TAD border and inter analyzed under C0, N1, and N2. Blue and dark-red represented TAD border and inter, respectively

While smaller than the A and B compartments, TADs are also key structural units that define chromosomal territories and are associated with the overall organization of the chromatin, with stronger chromatin interactions in TAD boundary regions as compared to TAD interior regions [22]. These observations have led researchers to posit that TADs comprise a principal component of overall chromosomal structure, partitioning the chromatin into distinct regions subject to autonomous regulation [35]. The insulation score algorithm [33] was successfully used to identify TAD-like structures within the *N. oceanica* genome (Fig. 3A; Chromosome 3 as an example), consistent with reports for maize and rice [19]. At a resolution of 2 kb, the C0, N1, and N2 samples were, respectively, found to harbor 1556, 1731, and 1797 TADs with a median length of 150 kb (Fig. 3B), covering ~ 52% of the *N. oceanica* genome. TAD boundary regions in these three respective samples contained 2252, 2484, and 2588 genes (Fig. 3C), and ND treatment was associated with the reorganization of many of these TADs (Fig. 3D). TAD transitions were initially classified into four previously described categories, including ‘Stable’ (TADs from two different treatments exhibit > 0.75 proportional overlap), ‘Split’ (one TAD separated into 2+ TADs), ‘Merge’ (2+ TADs merged into a single TAD), and ‘Rearrangement’ (all other cases) transitions [36]. For the C0 vs. N1 and C0 vs. N2 comparisons, 232 and 256 ‘Split’ TAD transitions and 344 and 394 ‘Merge’ TAD transitions, respectively, were evident at a resolution of 2 kb (Fig. 3D), suggesting that these TAD rearrangements may be associated with shifts in gene expression patterns in response to nitrogen deprivation. No significant differences in gene density or GC content were observed when comparing identified TAD boundary and interior regions within the *N. oceanica* genome (Additional file 1: Fig. S9A, B). Levels of gene expression, however, were significantly elevated in boundary regions as compared to interior regions in all three sample groups (Fig. 3E). The top 5 motifs that were enriched in these TAD boundary regions for the C0 vs. N1 and C0 vs. N2 comparisons were also identified (Additional file 1: Fig. S9C). The MA1281.1, MA1236.1, MA1226.1, and MA1278.1 motifs were shared in the C0, N1, and N2 samples, whereas the MA1274.1 motif observed in C0 samples was replaced by the MA1240.1 in both ND samples (Additional file 1: Fig. S9C). There were 349, 354 and 385 TAD borders shared between C0 and N1, or C0 and N2, or N1 and N2, respectively (Additional file 1: Fig. S10). Overall, changes were observed in roughly 21.8% and 22.9% of TAD regions for the C0 vs. N1 and C0 vs. N2 sample pairs, respectively, consistent with pronounced chromatin rearrangement under ND conditions.

**Fig. 4:**
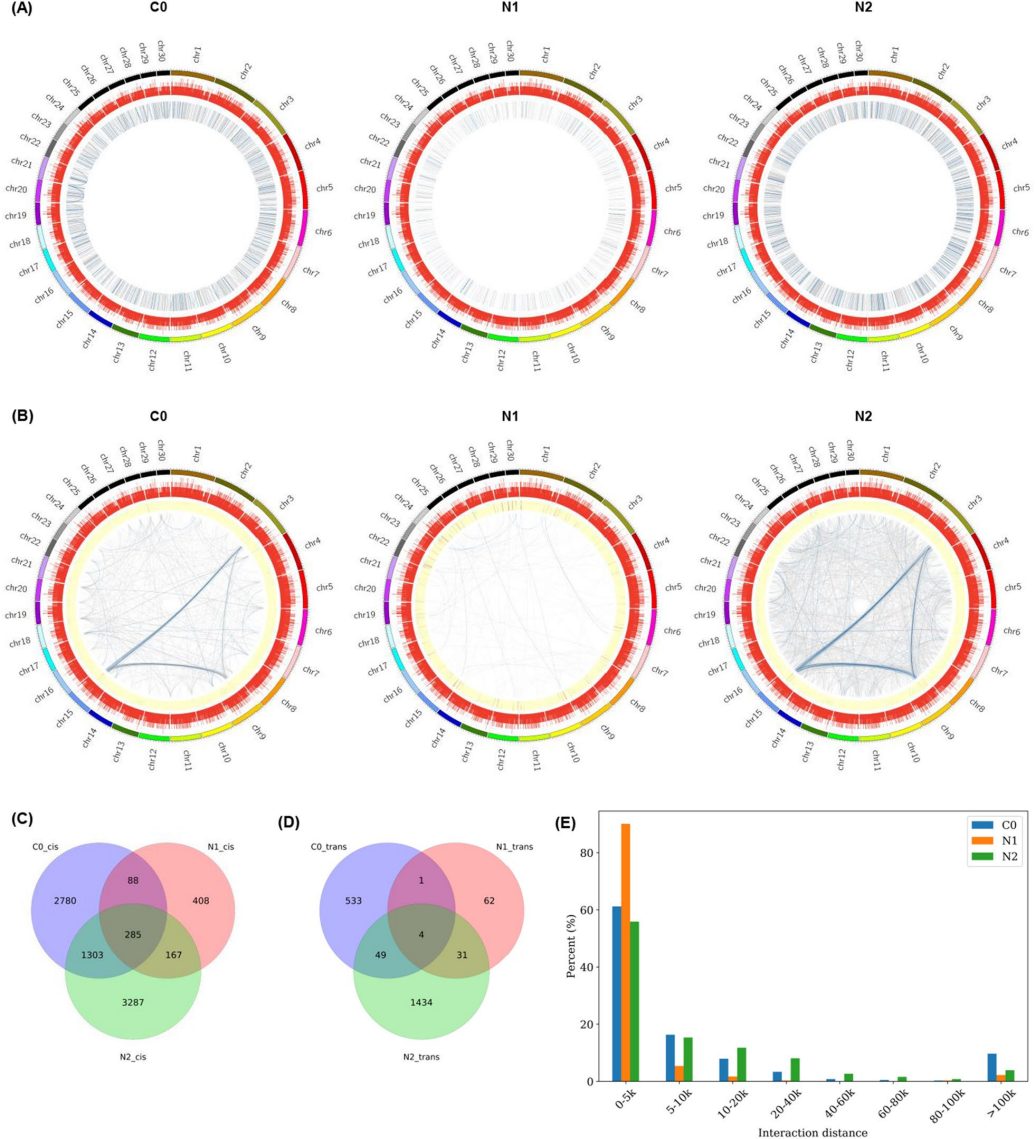
Chromatin interactions in *N. oceanica*. **A** Circos of genomic cis-interactions under C0, N1 and N2. **B** Circos of genomic trans-interactions under C0, N1 and N2. **C** Venn diagram of cis-interactions under C0, N1 and N2, respectively. **D** Venn diagram of trans-interactions under C0, N1 and N2, respectively. **E** Distribution of genomic distance of cis-interactions under C0, N1 and N2 in *N. oceanica*

Chromatin loops are relatively small chromatin structural units that form and enable distant genomic elements and transcriptional units to physically contact one another, thereby favoring transcriptional activation. The Juicer pipeline HiCCUPS algorithm [37] was next used to detect chromatin loops and the Fit-Hi-C [38] was employed to detect intra-chromosomal interactions (cis-interactions; Fig. 4A) and inter-chromosomal interactions (trans-interactions; Fig. 4B), revealing 4456, 948, and 5042 cis-interactions (Fig. 4C) and 587, 98, and 1518 trans-interactions (Fig. 4D) in the C0, N1, and N2 samples, respectively. Relative to previously reported data from higher plants (such as *Arabidopsis* and rice) and mammals [39], the numbers of chromatin loops identified within the *N. oceanica* genome were relatively low.

### Nitrogen deprivation induces correlated changes in chromatin reorganization and gene expression in *N. oceanica*

**Fig. 5:**
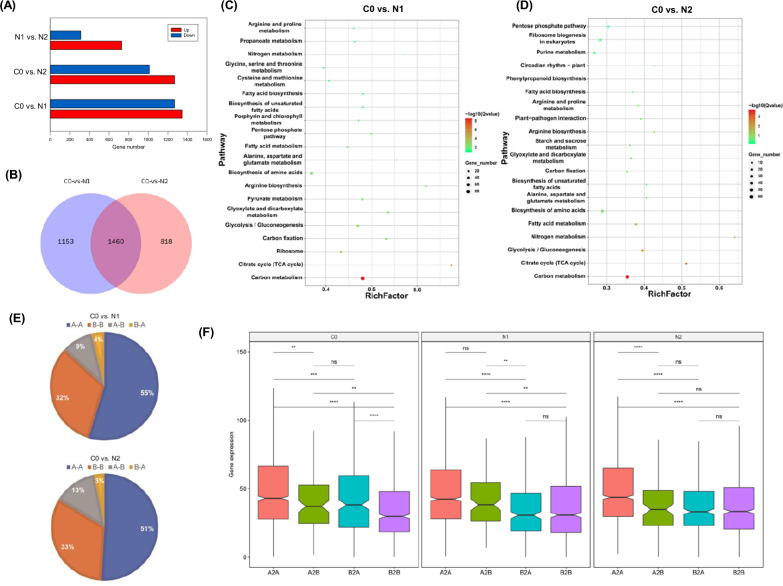
Correlation of chromatin 3D structure and gene expression under nitrogen deprivation in *N. oceanica*. **A** Comparison of the number of up- and downregulated genes under C0 vs. N1, C0 vs. N2, and N1 vs. N2, respectively. Fold change ≥ 2. **B** Venn diagram of overlapped DEGs under C0 vs. N1 and C0 vs. N2. **C**, **D** KEGG pathway enrichment analysis of DEGs under C0 vs. N1, C0 vs. N2, respectively. **E** Pie chart showed the percent of A/B compartment switching under C0 vs. N1 and C0 vs. N2. **F** Gene expression features of A/B compartments. The gene expression (FPKM value) of A compartments, A to B switching compartments, different compartments, B to A switching compartments, and B compartments under C0 vs. N1 and C0 vs. N2, respectively

Dynamic chromatin reorganization was evident in ND-treated *N. oceanica* cells, including both A/B compartmental switching and TAD transitions (Figs. 2 and 3). To explore changes in gene expression that may be associated with such genomic reorganization, mRNA-seq was next performed for two biological replicates for each of the three experimental groups (C0, N1, N2) (Additional file 1: Fig. S1A and “Methods”). This approach generated 41.83 Gb of mRNA-seq data (Additional file 2: Table S4), with strong correlations between biological replicate samples (Pearson correlation coefficient > 0.97; Additional file 1: Fig. S11A, B). In total, 2613 and 2278 differentially expressed genes (DEGs) were identified for the C0 vs. N1 and C0 vs. N2 comparisons, respectively, of which 1345 and 1268 were upregulated, while 1268 and 1010 were downregulated (|fold change| > 2, *P* < 0.01) under ND conditions*,* respectively (Fig. 5A, B). These DEGs included 1460 genes that were differentially expressed under both ND conditions relative to C0 control samples (Fig. 5B). The functional roles of these genes were explored through a KEGG pathway analysis, which revealed that these genes were enriched in carbon/nitrogen metabolism, fatty acid biosynthesis, pyruvate metabolism, biosynthesis of unsaturated fatty acid, fatty acid metabolism pathways for the C0 vs. N1 and C0 vs. N2 comparisons (Fig. 5C, D). Consistent with previous studies [8, 32], genes associated with TAG assembly and fatty acid metabolism pathways involved in lipid accumulation were upregulated in these *N. oceanica* cells in response to ND conditions, suggesting that the ability of cells to maintain appropriate energy metabolism can enable them to better adapt to nitrogen stress.

The analyses detailed above revealed higher levels of gene density and expression in A compartments relative to B compartments for both the C0 vs. N1 and C0 vs. N2 comparisons (Fig. 2C, E). To more fully explore how the 3D structural organization of the genome influences gene expression in the context of lipid accumulation, compartment A/B transitions were compared between NR and ND conditions. In total, 10,569 genes were identified in these *N. oceanica* cells across all three experimental conditions, with the majority of these genes being conserved in the A–A (55%) and B–B (32%) compartments (Fig. 5E). Just 13% of these genes were associated with A/B compartment shifts, including 9% and 4% that were, respectively, associated with A–B and B–A compartment switching when comparing C0 and N1 samples. Similarly, most of the detected genes were conserved in the A–A (51%) and B–B (33%) compartments when comparing C0 and N2 samples, while 13% and 3% were, respectively, associated with A–B and B–A compartment switching. The 2.6% of genes associated with A–B shifts from NR (C0) to ND (N1 or N2) conditions tended to be expressed at lower levels than those genes conserved in A compartments when comparing these *N. oceanica* sample groups (Fig. 5F). In contrast, genes associated with B–A shifts tended to be expressed at higher levels than genes conserved in B compartments (Fig. 5F). No significant differences in gene density or GC content were observed in the regions exhibiting A/B compartment switching (Additional file 1: Fig. S8). As for TAD, gene expression did not show significant difference in different transition types, including ‘stable’, ‘split’, ‘merge’, and ‘rearrangement’ under C0 vs. N1 or C0 vs. N2, respectively (Additional file 1: Fig. S12).

Comparisons of DEGs present within regions subject to A/B compartmental switching revealed that 243 and 225 genes in regions that underwent B–A compartment transitions were significantly upregulated in the C0 vs. N1 and C0 vs. N2 comparisons, respectively. Moreover, 693 and 847 genes in regions that underwent A–B compartment transitions were downregulated for these two respective sample comparisons. Functional enrichment analyses revealed that 13 and 12 lipid metabolism-related genes located in A/B transition regions were evident for the C0 vs. N1 and C0 vs. N2 comparisons, respectively (Additional file 1: Fig. S13A). In TAD border regions, some genes related to lipid metabolism or fatty acid biosynthesis were enriched (Additional file 1: Fig. S13B). Additionally, some genes related to signal transduction and carbohydrate metabolism were also enriched in these active regions. The observed 3D structural reorganization of the *N. oceanica* genome under ND conditions thus appears to be closely related to changes in lipid accumulation-related gene expression in this marine microalga.

### Chromatin 3D structural characteristics and histone modifications are correlated in the *N. oceanica* genome

To explore any potential correlative relationships between changes in the higher-order organization of the chromatin and histone modifications, a chromatin immunoprecipitation (ChIP)-seq analysis was next conducted to identify three different histone modifications (H3K27ac: Histone H3 lysine 27 acetylation; H3K36me2: histone H3 lysine 36 dimethylation; Kcr: lysine crotonylation; Additional file 2: Table S5). This approach offers insight into accessible regions of the genome, thereby facilitating analyses of active and inactive chromatin regions in these different *N. oceanica* samples. Two biological replicates per sample were used for ChIP-seq, with strong correlations between these replicate samples in the resultant dataset (Pearson correlation coefficient > 0.93; Additional file 1: Fig. S14), consistent with the reliable detection of these target chromatin modifications.

When analyzing histone modification distributions in these samples, Kcr and H3K27ac were found to be enriched in transcriptional start site (TSS) regions (Additional file 1: Fig. S15), whereas H3K36me2 was enriched in gene body regions (Additional file 1: Fig. S16). The overall enrichment patterns for the Kcr and H3K27ac modifications were largely similar, with levels of histone modification being, respectively, positively and negatively correlated with trans-interaction numbers and GC DNA content. Kcr and H3K27ac densities were both positively correlated with gene expression levels. In total, the C0, N1, and N2 samples were found to harbor 4327, 4315, and 3980 Kcr peaks, 4071, 3176, and 2579 H3K36me2 peaks, and 4416, 3737, and 3861 H3K27ac peaks, respectively (Additional file 2: Table S6). Of these, 3885, 3653, and 4025 overlapping Kcr, H3K36me2, and H3K27ac peaks, respectively, were observed when comparing C0 and N1 samples (Additional file 2: Table S7), while 4245, 2748, and 3950 overlapping Kcr, H3K36me2, and H3K27ac peaks were detected when comparing C0 and N2 samples (Additional file 2: Table S7).

**Fig. 6:**
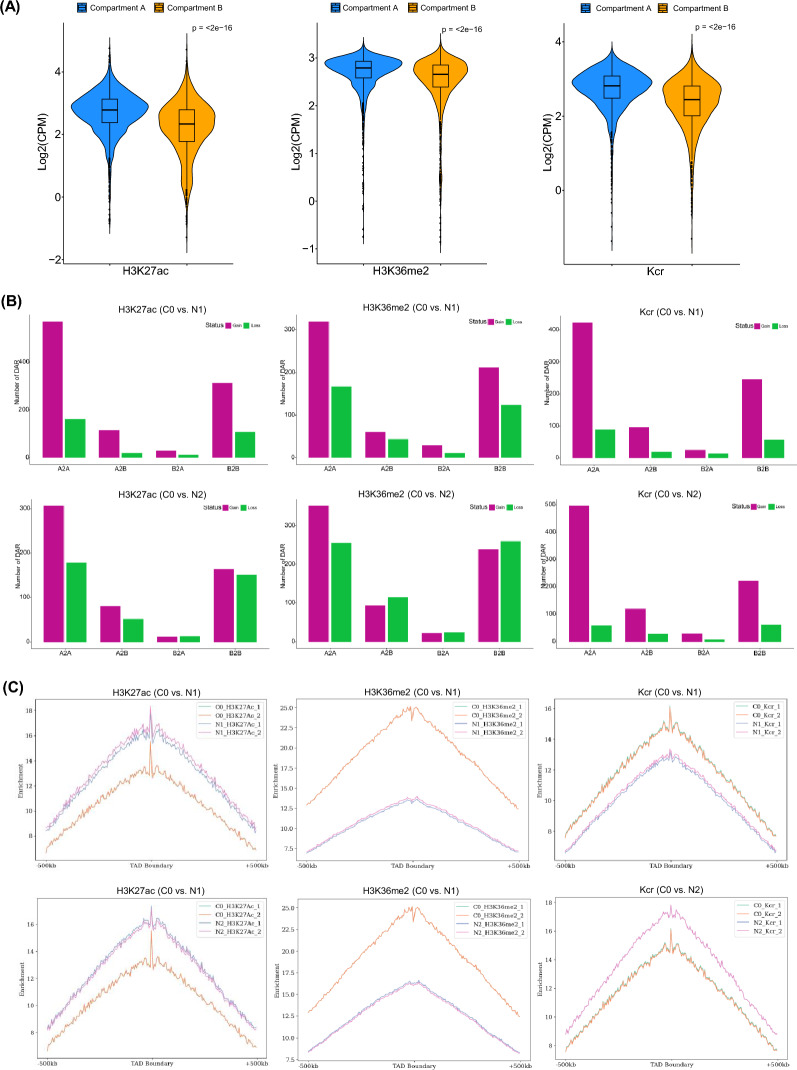
Chromatin 3D structure and histone modification are correlated upon nitrogen deprivation in *N. oceanica*. **A** Modification level of H3K27ac, H3K36me2 and Kcr in A and B compartments under C0. “***” indicates a significant difference at *P* < 0.001 using Wilcoxon unpaired test. **B** Distribution of gain or loss peaks of epigenetic marks (H3K27ac, H3K36me2 and Kcr) in the switching regions of compartment A and B in N. oceanica under C0 vs. N1, C0 vs. N2, and N1 vs. N2. **C** Enrichment of peaks of epigenetic marks (H3K27ac, H3K36me2 and Kcr) in the TAD boundary in *N. oceanica*

To more fully explore the associations between A/B compartment designations and histone modifications, the levels of these different epigenetic markers were next evaluated in these two genomic compartments. These analyses revealed that Kcr and H3K27ac levels tended to be higher in compartment A relative to compartment B (Fig. 6A). While H3K36me2 tended to be enriched in gene bodies, this modification was also more abundant in compartment A. These data support a link between the 3D structural organization of the chromatin and epigenetic modifications. Histone modification peak gains/losses were next compared in these compartments for the C0 vs. N1 and C0 vs. N2 sample comparisons, revealing 2504 peak gains and 2397 loss for these two respective sample comparisons. Most peak gains or losses were enriched in A–A or B–B transition regions, with relatively low levels of gains and losses in A–B and B–A transition regions (Fig. 6B).

To extend these results to other levels of chromatin structural organization, the relationship between TADs and histone modifications was further interrogated in these *N. oceanica* samples. In total, 2621 TADs with a median length of 105 kb were identified at 5-kb resolution, corresponding to ~ 45% of the *N. oceanica* genome (Fig. 2). TAD boundaries were associated with higher levels of H3K27ac, Kcr, and H3K36me2 modifications (Fig. 5B). Accordingly, both histone modifications and expressed genes were enriched in these boundary regions (Figs. 3E and 5C), indicating that the establishment of these TADs may coincide with the opening of the chromatin and active gene expression activity. These TAD boundaries were also enriched for some motifs (including C4 zinc finger-type factors [MA1281.1, MA1278.1 and MA1274.1), AP2/EREBP (MA1236.1, MA1226.1 and MA1240.1)], as has been reported in other species (Additional file 1: Fig. S9C; *E*-value < 4.7e−5), suggesting that proteins that interact with these motifs play a role in establishing TADs. There is thus a close relationship between epigenetic modifications and chromatin TAD structures.

### Nitrogen stress induces the reprogramming of lipid metabolism via chromatin structural reorganization and changes in patterns of histone modification

**Fig. 7:**
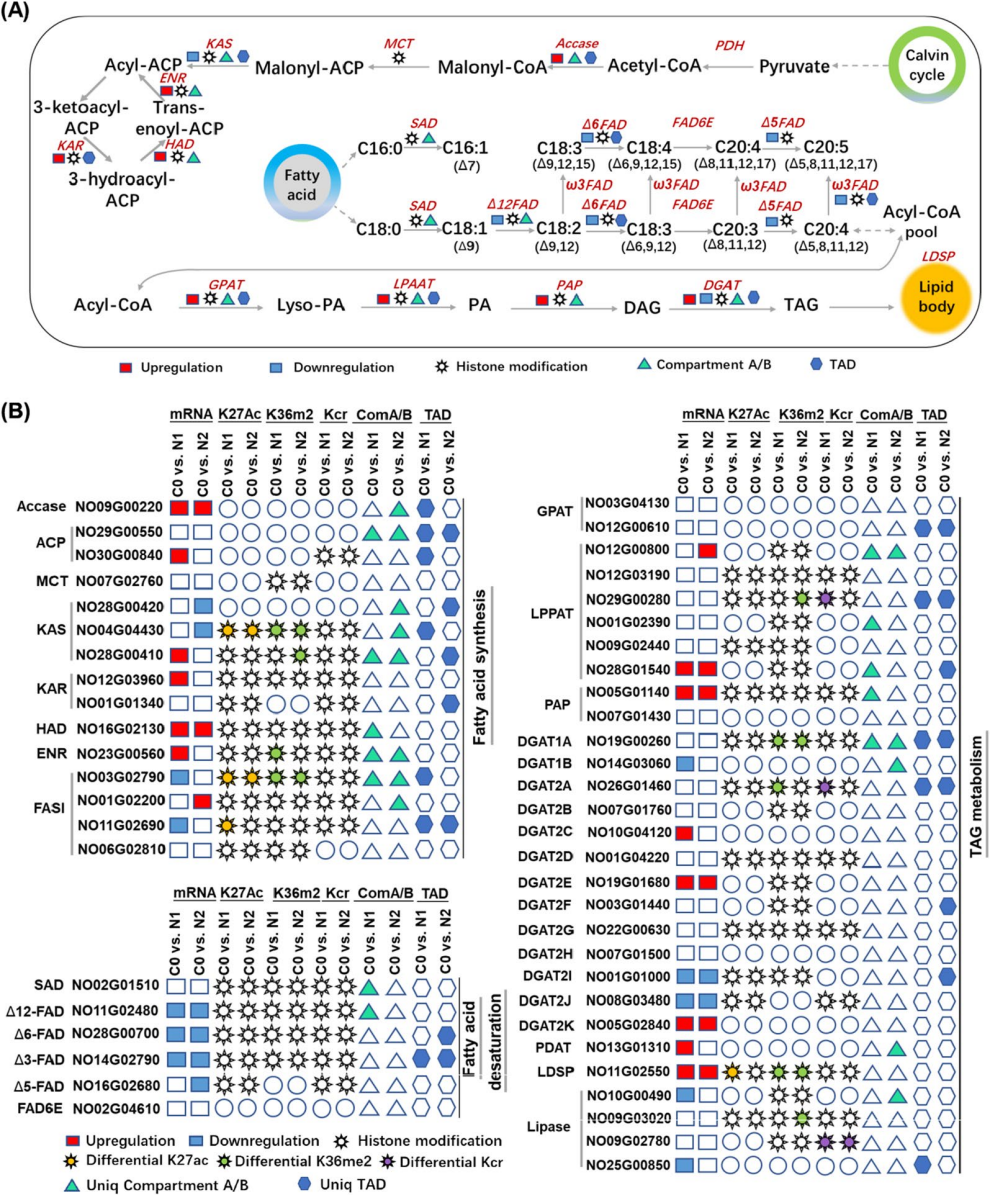
Reorganization of chromatin 3D architecture and epigenetic reprogramming of lipid metabolism for oil accumulation under nitrogen deprivation. **A** Schematic pathway of lipid metabolism. **B** Dynamic of gene expression, epigenetic marks and chromatin reorganization under C0 vs. N1 and C0 vs. N2 in *N. oceanica*. The upregulation or downregulation of genes, epigenetic signals of H3K27as, H3K36me2 and Kcr, dynamic of chromatin 3D organization (compartment and TAD) were shown under C0 vs. N1 and C0 vs. N2

Prior studies have documented the regulation of lipid accumulation in *N. oceanica* under nitrogen stress conditions at the transcriptional and protein levels [8, 29]. To extend these analyses, associations between ND-related changes in chromatin structural reorganization, histone modifications, and the lipid pathway were next explored in these microalgae. Acetyl-CoA is used as a precursor carbon source for de novo fatty acid synthesis by type II fatty acid biosynthesis pathway enzymes such as acetyl-CoA carboxylase (ACCase; NO09G00220), mitochondrial acyl carrier protein (ACP; NO29G00550 and NO30G00840), malonyl CoA-acyl carrier protein transacylase (MCT; NO07G02760), 3-ketoacyl-ACP synthase (KAS; NO04G04430, NO28G00410, and NO28G00420), and enoyl-ACP reductase (ENR; NO23G00560) (Fig. 7A), while type I fatty acid synthase (FASI) encodes a protein harboring functional domains closely related to fatty acid synthesis. Of these lipid metabolism-related genes, different compartmental assignments were evident in the 3D structure of *N. oceanica* chromatin for several of these genes including Accase (NO09G00220), ACP (NO29G00550), KAS (NO04G04430, NO28G00410, and NO28G00420), HAD (HAD: β-hydroxyacyl-acp dehydratase; NO16G02130), and ENR (NO23G00560) for the C0 vs. N1 or C0 vs. N2 comparisons. TADs were also associated with the Accase (NO09G00220), ACP (NO29G00550), and FASI (NO11G02690) genes for the C0 vs. N1 or C0 vs. N2 comparisons. Moreover, several of these genes were upregulated in ND samples (N1 or N2) relative to C0 control samples including Accase (NO09G00220), KAS (NO28G00410), HAD (NO16G02130), and ENR (NO23G00560), with such upregulation potentially being related to 3D chromatin reorganization (Fig. 7B). With respect to the three analyzed histone modifications, most analyzed fatty acid synthesis-related genes were associated with H3K27ac, H3K36me2, and Kcr modifications with the exception of Accase, ACP, and MCT. Differential H3K27ac and H3K36me2 peaks were also evident for KAS (NO04G04430) and FASI (NO03G00560) for both the C0 vs. N1 and C0 vs. N2 comparisons. The enzymes discussed above specifically play roles in de novo fatty acid synthesis and associated elongation, but *N. oceanica* cells are also capable of engaging a desaturation process to generate polyunsaturated fatty acids including eicosapentaenoic acid (EPA, C20:5) and docosahexaenoic acid (DHA, C22:6) [8, 40]. EPA, for example, is produced from the saturated fatty acid C18:0 through a series of enzymatic reactions mediated by stearoyl-ACP desaturase (SAD), Δ12 fatty acid desaturase (Δ12-FAD), Δ6-FAD, ω3-FAD, Δ6 fatty acid elongase (Δ6-FAE), and Δ5-FAD. Significant decreases in Δ3-FAD (NO14G02790), Δ5-FAD (NO16G02680), Δ6-FAD (NO28G00700), and Δ12-FAD (NO11G02480) expression were observed in both the N1 and N2 samples relative to C0 samples (Fig. 7). Strikingly, compartmental or TAD alterations were also observed for Δ12-FAD, Δ6-FAD, and Δ3-FAD when comparing control and ND samples, suggesting that fatty acid desaturation is also regulated by dynamic chromatin reorganization under conditions of nitrogen stress.

*Nannochloropsis* cells can achieve TAG biosynthesis through the acyl-CoA-dependent Kennedy pathway or through an alternative acyl-CoA-independent pathway [8, 40]. Glycerolipid assembly mediated through the Kennedy pathway relies on enzymes including glycerol-3-phosphate acyltransferase (GPAT), lysophosphatidic acid acyltransferase (LPAAT), phosphatidate phosphatase (PAP), diacylglycerol acyltransferase (DGAT) and lipid droplet surface protein (LDSP). Elevated GPAT (NO12G00800), LPPAT (NO28G01540), PAP (NO05G01140), and PDAT (NO13G01310) mRNA levels were observed under ND conditions with a corresponding shift in associated chromatin structural characteristics (Fig. 7). The 13 encoded DGATs also exhibited diverse regulatory changes under ND conditions, as reported previously [8]. Specifically, DGAT2E and DGAT2K were significantly upregulated under the C0 vs. N1 and C0 vs. N2 conditions, in line with prior results [8], although no associated changes in patterns of chromatin structural characteristics or histone modification were observed. Conversely, the DGAT1A and DGAT2A genes were associated with changes in associated chromatin compartments, TADs, and histone modification patterns under ND conditions relative to C0 conditions without any corresponding changes in mRNA levels (Fig. 7). LDSP (NO11G02550), however, exhibited a 4.2-fold increase in mRNA levels as well as differential H3K27ac and H3K36me2 peaks for both the C0 vs. N1 and C0 vs. N2 comparisons, although no associated shifts in chromatin compartments or TADs were detected for this gene (Fig. 7). Multiple putative TAG lipases (NO10G00490 and NO25G00850) also exhibited ND-induced downregulation at the mRNA level together with differential patterns of chromatin organization and histone modification.

Together, these data suggest that dynamic chromatin reorganization may govern fatty acid synthesis and TAG assembly in these microalgal cells. These changes in chromatin structural characteristics are also likely to impact the epigenetic state of the chromatin, consistent with the observed changes in H3K27ac, H3k36me2, and Kcr modification patterns associated with genes related to fatty acid synthesis and TAG assembly, suggesting that ND-induced lipid accumulation in *N. oceanica* is tightly regulated by the structural characteristics of the chromatin.

## Discussion

There has been growing interest in recent years in the development of *N. oceanica* as a model microalgal species for use in industrial settings. While *Nannochloropsis* species have thus far only been commercially leveraged for nutraceutical production or as a food source for aquaculture, they offer great potential as adaptable cells that can be leveraged to produce a wide variety of biological products. Depending on the conditions under which they are cultured, these rapidly growing microalgae can be rich sources of lipids or proteins produced at a commercial scale. Under nitrogen stress conditions, *Nannochloropsis* cells produce high levels of EPA and other desirable omega-3 fatty acids, which comprise ~ 60% of TAGs following such stress exposure. Accordingly, many studies have explored the regulation of lipid metabolism in *Nannochloropsis* spp. at the transcriptional and protein levels, establishing a complex and dynamic metabolic network, while generating a growing set of tools that can be used for the genetic manipulation of these microalgal cells. RNA-seq analyses of *N. oceanica* CCMP1779, *N. gaditana* CCMP526, and *N. oceanica* IMET1 aimed at exploring the transcriptional basis for ND-induced neutral lipid accumulation in *Nannochloropsis* cells have identified anywhere from 939 to 3255 genes subject to transcriptional reprogramming in response to nitrogen stress [8, 27–29]. In the present study, we expanded on these prior analyses by examining the role that chromatin structural reorganization and changes in histone modification patterns play in shaping nitrogen stress-related transcriptional responses in *N. oceanica.* These analyses are significant, given that this represents the first study to our knowledge focused on decoding the importance of the regulatory interplay between chromatin reorganization and transcriptional activity in these marine microalgae, introducing a new layer of understanding into the mechanistic model of the processes that control lipid accumulation under ND conditions.

Megabase-scale TADs separated by CCCTC-binding factor (CTCF) protein and cohesion complex-enriched regions are found throughout the mammalian genome [11]. The *Caenorhabditis elegans* genome consists of self-interacting TAD-like regions with strong boundaries owing to the absence of a CTCF insulator protein [41]. Similarly, plant species including *Arabidopsis*, peanut, soybean, and rice lack this CTCF insulator, yet their genomes nonetheless exhibit TAD-like domains and TAD boundary motifs [15, 22, 42, 43]. No CTCF protein homolog was detected in the *N. oceanica* genome in this study, but certain motifs (C4 zinc finger-type factors and AP2/EREBP) were present at TAD boundaries in the genome of this microalga, suggesting that these motifs may support TAD formation much as has been reported in higher plants. While mammalian cells often exhibit the conservation of TAD in the chromatin architecture, such conservation is less apparent in plants such as maize [16, 17], *Solanum lycopersicum* [19], *Sorghum bicolor* [19], foxtail millet (*Setaria italica*), and rice. Strikingly, *N. oceanica* cells exposed to ND conditions exhibited clear variations in TAD patterns in response to environmental stress (Fig. 5). Of the four TAD transition types (‘Stable’, ‘Split’, ‘Merge’ and ‘Rearrangement’) defined in a prior study [36], ND was associated with 232 and 256 ‘Split’ TAD transitions as well as 344 and 394 ‘Merge’ TAD transitions for the respective C0 vs. N1 and C0 vs. N2 comparisons (Fig. 3C). TADs have been reported to be subject to complex shifts in response to changes in environmental conditions in other species. For example, Drosophila Kc167 cells exposed to heat shock conditions exhibit rapid TAD reorganization mediated by the relocation of architectural proteins from TAD boundaries to interior regions [44]. Environmental stressors may thus shape the distribution of TADs and their structural evolution. Associations between TAD transitions and gene expression levels in ND-exposed *N. oceanica* cells were further explored to better understand the regulatory implications of these transitions. However, no differences in gene expression levels were associated with these TAD transitions (Additional file 1: Fig. S12), indicating that these transitions may play distinct regulatory roles in these microalgal cells.

In other species, TAD boundaries often are strongly correlated with local differences in gene expression, epigenetic landscape, and the presence of various insulator proteins [34]. As genes proximal to TAD boundaries were generally expressed at higher levels in this study (Fig. 3E), we further assessed the distributions of the epigenetic markers H3K27ac, H3K36me2, and Kcr around these boundary regions. In line with the observed enrichment of highly expressed genes in these TAD boundary regions, similar enrichment of H3K27ac, H3K36me2, and Kcr, which are associated with active chromatin, was also evident (Fig. 6C). The H3K27ac and Kcr histone markers, which are primarily associated with transcriptional start sites, were symmetrically distributed at these TAD boundaries (Fig. 6C). However, H3K36me2 was not symmetrically distributed relative to these TAD borders, potentially because this modification was frequently enriched in gene body regions, which are more abundant in regions outside of the defined TADs. The H3K27ac and Kcr modification is associated with active chromatin and gene expression in *N. oceanica*, supporting a model in which preferential transcription occurs at TAD boundary regions. Overall, these results support a link between TAD boundaries and active transcriptional activity such that this is likely an intrinsic characteristic of TAD boundary regions.

When analyzing the high-level structural properties of the *N. oceanica* genome, A and B compartments, respectively, exhibiting positive and negative eigenvectors corresponding to active and inactive regions of the genome were successfully identified (Fig. 2A), much as has been reported in other studies of plant and mammalian genomes. In these microalgal cells, the A compartment comprised a larger percentage of the genome than the B compartment. A/B compartment switching is commonly observed in plant cells exposed to environmental stimuli. For example, one study employing eigenvalue analyses revealed that the first and second B compartments surrounded by A compartments in the rice genome under normal conditions could undergo conversion into A compartments, yielding a substantially larger A compartment under heat stress [23]. When rice seedlings were exposed to a range of temperatures, researchers generated chromatin interaction maps demonstrating that while global chromatin organization remained stable under these conditions, cold stress was associated with the smaller local-scale decondensation of chromosomes in these seedlings [22]. A/B compartmental switching was also evident in *N. oceanica* cells in the present study (Additional file 1: Fig. S7). Heat stress can induce transposon activity in *Arabidopsis* cells, thereby supporting the global rearrangement of the genome at the 3D structural level [21], with heat-induced transitions (A–B or B–A) being evident for approximately 7% of A/B compartments. Just 3.7% A/B compartment switching, however, was evident in domesticated soybeans [43]. Approximately 13–16% of A/B compartments in *N. oceanica* underwent transitions in this study following ND exposure, with this percentage being higher than the values reported in other species. A/B compartment transitions tend to be related to altered transcription activity, with B–A and A–B transitions most often being associated with gene downregulation and upregulation, respectively [22, 45]. Consistently, significant differences in gene expression for regions exhibiting A–B or B–A transitions in the *N. oceanica* genome were observed, whereas gene expression for A–A or B–B regions remained stable (Fig. 5F). With respect to the relationship between these chromosomal compartments and epigenetic modification patterns, higher levels of the active H3K27ac and Kcr chromatin markers were observed in the A compartment in these *N. oceanica* cells (Fig. 6A). Similar A/B compartment patterns have also been reported in *Arabidopsis* [15], rice [22], and *M. truncatula* [24]. In cotton, active regions bearing H3K27ac modifications were also more likely to be observed in the A compartment [45]. Under ND conditions, transcriptional changes in *N. oceanica* were thus found to be associated with both chromatin structural reorganization and histone modification patterns, underscoring the role that dynamic changes in the architectural characteristics of the chromatin play in shaping transcriptional responses to environmental stimuli.

In this study, clear evidence was observed that lipid metabolism reprogramming is under the dynamic control of chromatin structural organization, including A/B compartments, TADs, and chromosome interactions, as well as histone modifications in *N. oceanica* cells under ND conditions. The observed 3D reorganization of the chromatin also exhibited time-dependent regulatory dynamics. GPAT (NO12G00800), LPPAT (NO28G01540), PAP (NO05G01140), and PDAT (NO13G01310) were all found to be upregulated at the mRNA level in *N. oceanica* cells exposed to nitrogen stress, and these genes were all associated with underlying chromatin structural changes, supporting a putative link between the chromatin architecture and transcriptional activity. Diverse expression patterns for DGATs were also observed, with DGAT1A and DGAT2A exhibiting pronounced differences in compartmental segregation and histone modification for the C0 vs. N1 and C0 vs. N2 comparisons despite no apparent differences in their expression at the transcriptional level (Fig. 7). A 4.2-fold increase in mRNA expression was also observed for the lipid droplet surface protein (LDSP; NO11G02550), which was further associated with differential H3K27ac and H3K36me2 peaks. These findings support a model in which *N. oceanica* lipid metabolism is subject to multi-level chromatin, epigenetic, and transcriptional regulation, suggesting the potential importance of multidimensional approaches to modulating lipid biosynthetic activity by genetic tools in future studies.

## Conclusions

In conclusion, this study is the first to have comprehensively integrated Hi-C, ChIP-seq, and RNA-seq datasets from *N. oceanica* IMET1, providing insight into local and global chromatin structural organization in cells deprived of sufficient nitrogen. These analyses demonstrated that nitrogen stress influenced A/B compartment transitions, TAD types, and long-range intra- and inter-chromosomal interactions within these microalgal cells. These changes in the 3D organization of the chromatin were also associated with pronounced changes in patterns of gene expression. Importantly, these data provide novel evidence for the role that chromatin structural reorganization and histone modifications play in the regulation of lipid metabolism, offering a foundation for future efforts to develop new approaches to enhancing or otherwise modulating microalgal lipid production. Overall, the distinct patterns of chromatin organization and associated shifts in transcriptional activity when comparing nitrogen-sufficient and nitrogen-deficient conditions provide fundamental evidence regarding the important role that chromatin structural shifts and epigenetic mechanisms play in shaping stress responses in microalgae.

## Methods

### Culture of *Nannochloropsis oceanica*

*Nannochloropsis oceanica* IMET1 was inoculated into modified f/2 liquid medium containing 35 g L^−1^ sea salt (Realocean, USA), 1 g L^−1^ NaNO_3_, 67 mg L^−1^ NaH_2_PO_4_*H_2_O, 3.65 mg L^−1^ FeCl_3_*6H_2_O, 4.37 mg l^−1^ Na_2_EDTA*2H_2_O, trace metal mix (0.0196 mg L^−1^ CuSO_4_*5H_2_O, 0.0126 mg L^−1^ NaMoO_4_*2H_2_O, 0.044 mg L^−1^ ZnSO_4_*7H_2_O, 0.01 mg L^−1^ CoCl_2_, and 0.36 mg L^−1^ MnCl_2_*4H_2_O), and vitamin mix (2.5 µg L^−1^ VB_12_, 2.5 µg L^−1^ biotin, and 0.5 µg L^−1^ thiamine HCl) as reported previously [46]. Cells were initially cultured in this medium in a 1 L column reactor with a 5 cm internal diameter at 25 °C while exposed to continuous illumination (80 ± 5 μmol m^−2^ s^−1^). *N. oceanica* cells were bubbled with air (0.04% CO_2_). When cells had reached the logarithmic phase of growth (OD_750_ = 2.0), they were collected via centrifugation for control sample and subsequent experiment. One part as a control (C0) sample (three replicates) was harvested for RNA and chromatin extraction, the other part (six replicate samples) was rinsed three times with fresh nitrogen-free media prior to subsequent experimental use. Cell pellets were suspended in nitrogen-free f/2 medium for mRNA-seq, ChIP-seq, and HiC-seq sample collection, or in nitrogen-replete f/2 medium when conducting physiological measurements. A starting concentration of OD_750_ = 2.0 was used for all cultures, which were continuously illuminated (50 ± 5 μmol m^−2^ s^−1^). Aliquoted cell samples were transferred to appropriate experimental conditions and collected as nitrogen-deprived samples after 1 or 2 days (N1 and N2; Algal cells were sampled at 24 h and 48 h after nitrogen-deprived cultivation) for mRNA-seq, ChIP-seq, and HiC-seq analyses, with three replicate samples for each experimental condition (Additional file 1: Fig. S1A). Previous studies have demonstrated that algal cells launched lipid accumulation at these two timepoints [8, 31, 32, 47].

### Physiological measurements

Chlorophyll fluorescence can be analyzed as a sensitive indicator of the instantaneous photosynthetic state of microalgae and their responses to environmental changes [48]. The variable/maximum fluorescence ratio (Fv/Fm) corresponds to the maximal PSII reaction center photochemical quantum yield, representing the minimum fluorescence yield when these reactions centers are fully open and associated photosynthetic efficiency such that this parameter is commonly used as a metric to gauge photosynthetic performance and acclimation. Fm represents the maximum fluorescence yield when PSII reaction centers are fully closed, corresponding to PSII electron transport capacity, while Fv represents the variable fluorescence (Fv = Fm − Fo) associated with the reduction of the PSII primary electron acceptor QA, thereby representing PSII reaction center photochemical activity. Fo represents the minimum fluorescence yield, which decreases with damage or other causes of the irreversible disruption of PSII reaction centers. The formula used to assess the Fv/Fm ratio was: Fv/Fm = (Fm − Fo)/Fm [48]. These parameters were measured by incubating *N. oceanica* samples for 20 min in the dark, followed by a 1 s exposure to a saturating pulse of light (1000 mol m^−2^ s^−1^) while measuring chlorophyll fluorescence intensity values based on pulse amplitude-modulated (PAM) kinetics with an imaging-PAM M-Series instrument (Walz, Germany) based on provided directions. A modified extraction method was used to measure Chl *a* content [49]. Briefly, algal culture samples were centrifuged at 2500×*g*, followed by extraction for 24 h in the dark using 100% ethanol saturated with MgCO_3_. After subsequent centrifugation, supernatants were subjected to spectrophotometric analysis, with a UV–Vis spectrophotometer being used to record extinction values at 632, 649, 665, and 750 nm. The oxygen evolution rate was determined with a Clark-type 2 oxygen electrode (Hansatech, UK). Chl *a* content in these *N. oceanica* cultures was used to normalize the rates of oxygen evolution.

### Hi-C experiments and sequencing

Hi-C analyses were performed by grinding samples of *N. oceanica* into a powder under liquid nitrogen, after which chromatin was extracted as in prior studies [50]. Then, 200 U of MboI (NEB) was utilized to digest genomic DNA for 4 h at 37 °C, followed by the use of biotinylated cytosine nucleotides to label the resultant restriction fragment ends with biotin-14-dCTP (NEB). Samples were then incubated with T4 DNA ligase (50 U) overnight at 16 °C for blunt end ligation, followed by overnight incubation with proteinase K (200 µg/mL, Thermo Fisher) at 65 °C [42]. DNA was subsequently purified with a QIAamp DNA Mini Kit (Qiagen), followed by the shearing of the resultant DNA into ~ 400 bp fragments with a Covaris M220 instrument. Ligation junctions were then precipitated using Dynabeads® MyOne™ Streptavidin C1 (Thermo Fisher). An Illumina TruSeq DNA Sample Prep Kit was then used for library preparation, followed by the use of an Illumina HiSeq 3000 instrument for 150 bp paired-end sequencing.

### Hi-C read processing

HiC-Pro was used for the processing of clean Hi-C data with a minimum 4000 bp cis distance. Contact maps were generated at a range of bin sizes (5, 10, 20, 40, and 100 kb) and converted to produce hic files with HiC-Pro scripts (10.1016/j.cels.2016.07.002). Chromosome-scale heatmaps were produced using HiCPlotter, and the HiCCUPS program was used to identify chromatin loops (parameters: -m 1024 -r 5000,10000 -k VC_SQRT -f 0.001,0.001 -p 4,2 -i 7,5 -t 0.02,1.5,1.75,2 -d 20000,20000). Custom scripts were utilized to merge significant interactions in replicate samples, while edgeR was used to identify differential interactions with a quasi-likelihood method and a *p*-value of 0.2. Sushi was used to visualize the identified interactions combined with other genomic datasets.

### Contact map construction

Hi-C data were subjected to quality control filtering with Trimmomatic (v 0.39) [51], after which clean data from two biological replicates from particular treatments or timepoints (C0, N1, and N2) were mapped to the genome (http://nandesyn.single-cell.cn/download;V2) [52] with the ICE software package (v 1f8815d0cc9e) [53]. Unusable data such as dangling ends were filtered out, while valid pairs were assessed to gauge correlation coefficients between the two biological replicates with GenomeDISCO [54], and the data from the two replicates were then pooled for downstream analyses. Following pooling, valid pairs were binned into non-overlapping (100 kb, 40 kb, 20 kb, 10 kb, 5 kb) genomic intervals for contact map generation.

### *Z*-score calculations and subtraction matrix construction

Overall chromatin interaction decay with distance was evaluated with a modified LOWESS method (alpha = 0.5%, ignore zeros, IQR filter) as in prior reports 30. This approach pools genome-wide data and calculates weighted average and weighted standard deviation values for all genomic distances. Interaction data were transformed into *Z*-scores as follows: [(observed signal − LOWESS-average)/LOWESS-stdev]. Signals were excluded from such transformation into *Z*-scores if they exhibited a count of 0. *Z*-scores were used as a means of correcting for small variations in the overall decay with genomic distance that may arise among samples.

### Compartment A/B analyses of Hi-C data

In the 40-kb bin-based single chromosome contact heatmaps, a plaid-like pattern is visible, with the alternating regions of high- and low-frequency interactions corresponding to compartments A and B. These compartments were identified at a 100-kb resolution with the CscoreTool (v 1.1) software [55]. Genomic bins with positive and negative *C*-score signals were divided into an active A compartment consisting of gene-dense regions of euchromatin and an inactive B compartment consisting of regions of gene-poor heterochromatin.

### TAD analyses of Hi-C data

TADs are contiguous genomic regions with a high degree of self-association and that have distinct boundaries separating them from nearby genomic regions. TAD locations can be defined when binning interaction data at 20 kb. For this study, TAD boundaries in individual samples were assessed with an insulation score algorithm [33] (arguments: --is100000 --ids 40000), and numbers and locations of TADs were additionally assessed.

### Intra- and inter-chromosomal interaction detection of Hi-C data

Intra- and inter-chromosomal interactions for each sample were assessed based on the contact between 10-kb bins using the Fit-Hi-C software (v2.0.8) with the settings: -L 10,000 -p2) [38]. This approach was used to compute corresponding cumulative probability *P*-values and false discovery rate (FDR) *q*-values. Interactions with a contact count > 2, a *p*-value < 0.01, and a *q*-value < 0.01 were considered significant.

### ChIP-seq library preparation and data analysis

*N. oceanica* (1–2 million cells mL^−1^) from an 800 mL culture were fixed at room temperature using 1% formaldehyde (Sigma) under vacuum for 30 min, after which they were treated with 0.15 M glycine under vacuum for 10 min at room temperature. Nuclei were then isolated as reported previously, with each round of ChIP being performed with the nuclei isolated from fixed material [50]. Briefly, after isolation, these nuclei were suspended in 1 mL of sonication buffer (10 mM potassium phosphate, pH 7.0, 0.1 mM NaCl, 0.5% sarkosyl, 10 mM EDTA), after which a Covaris S220 instrument was used to shear genomic DNA to an average 300–1000 bp fragment size. This ample was then combined with 100 µL 10% Triton X-100, with 50 µL of the resultant sample being saved as an input sample and the remainder being mixed with an equivalent amount of IP buffer (50 mM Hepes, pH 7.5, 150 mM NaCl, 5 mM MgCl_2_, 10 µM ZnSO_4_, 1% Triton X-100, 0.05% SDS) followed by incubation overnight with anti-H3K36me2 (Abcam, ab9049), anti-H3K27Ac (Abcam ab4729), or anti-Kcr (PTM Biolabs, PTM502) at 4 °C. Then, samples were mixed with 50 µL of Protein A/G magnetic beads (Millipore) followed by a 2 h incubation at 4 °C. Beads were subsequently washed (5 min/wash) three times using IP buffer, one time using IP buffer supplemented with NaCl (500 mM), and one time with LiCl buffer (0.25 M LiCl, 1% NP-40, 1% deoxycholate, 1 mM EDTA, 10 mM Tris pH 8.0). Then, 200 µL of elution buffer (50 mM Tris, pH 8.0, 200 mM NaCl, 1% SDS, 10 mM EDTA) was incubated with chromatin-bound beads for 30 min at 65 °C, after which samples were treated for 6 h with Proteinase K at 65 °C. A Qiagen kit (Qiagen) was subsequently used to extract DNA from these samples, which were then subjected to end repair, A-tailing, adaptor ligation, and library amplification based on standard protocols (Illumina). An Illumina HiSeq 2500 instrument was then used to sequence (1 × 150 bp) the resultant library, with a slightly modified version of the nfcore/chipseq pipeline [56] being used to analyze the obtained ChIP-Seq data. Briefly, Trim Galore! was utilized to trim reads (https://www.bioinformatics.babraham.ac.uk/projects/trim_galore/), followed by alignment with BWA [57] to the *N. oceanica* reference genome. Picard was used to mark duplicate mapped reads, which were analyzed with DeepTools [58] and MACS2 [59]. Peak calling was performed with the “--broad” flag, and input sample reads served as controls. Additionally, diffReps [60] was used for differential peak analyses, with the ChIPpeakAnno [61] package then being utilized to annotate these differential peaks.

### RNA-seq analyses

Cells were centrifuged at 2500×*g* for 5 min, snap-frozen with liquid nitrogen, and stored at − 80 °C for further analysis. Trizol (Invitrogen, USA) was used to extract total RNA from these samples, after which a NanoDrop-1000 instrument (Thermo Scientific, USA) was used to evaluate RNA sample concentrations and purity. Sera-mag Magnetic Oligo (dT) Beads (Thermo Scientific, USA) were used to purify poly-adenylated mRNAs from these samples, followed by incubation in an RNA Fragmentation Reagent (Ambion, USA) to generate 200–300 bp fragments based on provided directions. Agencourt RNA Clean beads (Beckman Coulter, USA) were then used to purify these fragmented RNA sequences, which were used for cDNA synthesis with the SuperScript Double-Stranded cDNA Synthesis Kit (Invitrogen, USA) using random hexamer primers. A NEBNext® mRNA Library Prep Reagent Set (New England Biolabs, USA) was then used for strand-nonspecific library preparation, followed by 2 × 150 bp paired-end sequencing with an Illumina HiSeq2000 instrument. Raw data were deposited in the NCBI GEO database with the accession number GSE225079.

The nfcore/rnaseq pipeline was used for analyses of RNA-seq results [62]. Briefly, raw (2 × 150 paired-end read data were subjected to quality control with TrimGalore! (https://www.bioinformatics.babraham.ac.uk/projects/trim_galore/), with remaining high-quality reads then being aligned to the *N. Oceanica* IMET1 genome (http://nandesyn.single-cell.cn/download) using STAR [63], with Picard being used to mark duplicate reads. Expression levels were subsequently quantified with featureCounts [64] and StringTie [65]. Gene expression levels were measured based on the number of reads aligned to annotated genes by featureCounts [64], with normalization to CPM, FPKM, and TPM values. Raw featureCounts-derived counts were additionally utilized to conduct differential analyses of gene expression performed using edgeR (R package) [66]. Significantly differentially expressed genes were those exhibiting a minimum of a twofold change in gene expression and an FDR-corrected *P*-value ≤ 1 when comparing control and ND conditions, with CPM values for at least two samples ≥ 1.

### Statistical analysis

The data were analyzed via one-way ANOVA with a least significant difference test.

### Supplementary Information

**Additional file 1: Figure S1.** Experimental design and physiological measurement under N repletion and deprivation. (**A**) Experimental design for Hi-C, ChIP-seq and mRNA-seq data collection. (**B**) OD750, photosynthetic parameter (Fv/Fm) and photosynthetic oxygen evolution (POE) under nitrogen repletion and deprivation. **Figure S2.** Depth in different resolutions. The matrix resolution of a Hi-C map was defined as the smallest locus size such that 80% of loci have at least 1000 contacts. The resolution of > 2 kb is suitable for following analysis. **Figure S3.** Minus heatmap matrix of each chromosome in response to nitrogen deprivation between C0 and N1. At 2kb resolution, subtraction heatmap matrix of each chromosome was constructed between C0 and N1. **Figure S4.** Minus heatmap matrix of each chromsome in response to nitrogen deprivation between C0 and N2. At 2kb resolution, subtraction heatmap matrix of each chromosome was constructed between C0 and N2. **Figure S5.** Dekay curve of interaction frequencies against increasing genomic distance for each chromosome in *N. oceanica*. The genomic bin size is 100kb. **Figure S6.** Gene density and GC content in the compartment A/B under C0 vs. N1 and C0 vs. N2. (**A**) GC content in the region of compartment A and B under C0, N1 and N2. (**B**) Gene density in the region of compartment A and B under C0, N1 and N2. (**C**) Comparison of compartment length in the region of compartment A and B under C0, N1 and N2. **Figure S7.** Genome eigenvector analysis of compartment A/B in chromosome 23, 26 and 30. Segregation of local A/B compartments using eigenvector under C0, N1 and N2 for chromosome 23, 26 and 30 in *N. oceanica*. Blue and dark-red represented compartment A and B, respectively. **Figure S8.** Gene density and GC content in the transition region of compartment A/B under C0 vs. N1 and C0 vs. N2.** A** GC content in the compartment A/B switching region under C0 vs. N1 and C0 vs. N2. **B** Gene density in the compartment A/B switching region under C0 vs. N1 and C0 vs. N2. **Figure S9.** Gene density and GC content in the region of TAD border and inter under C0 vs. N1 and C0 vs. N2. (**A**) GC content in the region of TAD border and inter under C0, N1 and N2. (**B**) Gene density in the region of TAD border and inter under C0, N1 and N2. (**C**) The top five motifs in the region of TAD border C0, N1 and N2. **Figure S10.** Venn diagram of TAD border between C0 and N1, or C0 and N2, or N1 and N2. The number of TAD border were compared between C0 and N1, or C0 and N2, or N1 and N2. **Figure S11.** Pearson correlation relationships of mRNA-Seq libraries. (**A**) Pearson correlation relationships of six mRNA-Seq libraries. (**B**) PCA analysis of six sequencing samples. **Figure S12.** Comparsion of the gene expression in TAD transition regions under C0 vs. N1 and C0 vs. N2. (**A**) and (**B**) showed gene expression level in TAD transition regions under C0 vs. N1 and C0 vs. N2, respectively. **Figure S13.** KEGG functional enrichment of up-regulated genes in compartment A/B and TAD under C0 vs. N1 and C0 vs. N2. (**A**) and (**B**) KEGG functional enrichment of up-regulated genes in compartment A/B under C0 vs. N1 and C0 vs. N2. (**C**) and (**D**) KEGG functional enrichment of up-regulated genes in the TAD bonder under C0 vs. N1 and C0 vs. N2. **Figure S14.** Correlation coefficients of ChIP-seq libraries. Heatmap showed correlation of different samples. **Figure S15.** Distribution of enrichment analysis in histone modifications. Histone modification enrichment profile across genes were showed. **Figure S16.** Pattern of histone modifications over 6kb regions surrounding the TSS for H3K27ac and H3K36me2.

**Additional file 2: Table S1.** Statistics of Hi-C sequencing data. **Table S2.** Percent of the genomic regions in compartment A/B. **Table S3.** Statistics of stable and switch compartment A/B. **Table S4.** Statistics of mRNA sequencing data. **Table S5.** Statistics of ChIP sequencing data. **Table S6.** Static of total peaks in three modifications (H3K27ac, H3K36me2 and Kcr). **Table S7.** Statistics of overlap peaks for H3K27ac, H3K36me2 and Kcr between C0 and N1, or C0 and N2.

## Data Availability

All data generated or analyzed during this study are included in its files. For mRNA sequencing, raw sequencing data are available at NCBI under accession GSE225079. For HiC sequencing, raw data are deposited at NCBI under GEO accession GSE223130. For ChIP-seq, raw data are deposited at NCBI under GEO accession GSE224086. The genome assembly and annotation results can be downloaded from the new established genome database (http://nandesyn.single-cell.cn/download) [52].

## Abbreviations

TAD: Topologically associating domain
ND: Nitrogen deprivation
DEGs: Differentially expressed genes
POE: Photosynthetic oxygen evolution
TAG: Triacylglycerol
H3K27ac: Histone H3 lysine 27 acetylation
H3K36me2: Histone H3 lysine 36 dimethylation
Kcr: Lysine crotonylation
TSS: Transcriptional start site
ACCase: Acetyl-CoA carboxylase
ACP: Mitochondrial acyl carrier protein
MCT: Malonyl CoA-acyl carrier protein transacylase
KAS: 3-Ketoacyl-ACP synthase
HAD: β-Hydroxyacyl-acp dehydratase
ENR: Enoyl-ACP reductase
FASI: Type I fatty acid synthase
EPA: Eicosapentaenoic acid
DHA: Docosahexaenoic acid
SAD: Stearoyl-ACP desaturase
FAD: Fatty acid desaturase
FAE: Fatty acid elongase
GPAT: Glycerol-3-phosphate acyltransferase
LPAAT: Lysophosphatidic acid acyltransferase
PAP: Phosphatidate phosphatase
DGAT: Diacylglycerol acyltransferase
LDSP: Lipid droplet surface protein
CTCF: CCCTC-binding factor
