# Supplementary material for: Remodeling of the 3D chromatin architecture in the marine microalga Nannochloropsis oceanica during lipid accumulation
Source: Biotechnol Biofuels Bioprod. 2023 Aug 17;16:129. doi: 10.1186/s13068-023-02378-0 (PMC10436460; doi:10.1186/s13068-023-02378-0)
Supplement: Supplementary file 1 — Additional file 1: Figure S1. Experimental design and physiological measurement under N repletion and deprivation. (A) Experimental design for Hi-C, ChIP-seq and mRNA-seq data collection. (B) OD750, photosynthetic parameter (Fv/Fm) and photosynthetic oxygen evolution (POE) under nitrogen repletion and deprivation. Figure S2. Depth in different resolutions. The matrix resolution of a Hi-C map was defined as the smallest locus size such that 80% of loci have at least 1000 contacts. The resolution of > 2 kb is suitable for following analysis. Figure S3. Minus heatmap matrix of each chromosome in response to nitrogen deprivation between C0 and N1. At 2kb resolution, subtraction heatmap matrix of each chromosome was constructed between C0 and N1. Figure S4. Minus heatmap matrix of each chromsome in response to nitrogen deprivation between C0 and N2. At 2kb resolution, subtraction heatmap matrix of each chromosome was constructed between C0 and N2. Figure S5. Dekay curve of interaction frequencies against increasing genomic distance for each chromosome in N. oceanica. The genomic bin size is 100kb. Figure S6. Gene density and GC content in the compartment A/B under C0 vs. N1 and C0 vs. N2. (A) GC content in the region of compartment A and B under C0, N1 and N2. (B) Gene density in the region of compartment A and B under C0, N1 and N2. (C) Comparison of compartment length in the region of compartment A and B under C0, N1 and N2. Figure S7. Genome eigenvector analysis of compartment A/B in chromosome 23, 26 and 30. Segregation of local A/B compartments using eigenvector under C0, N1 and N2 for chromosome 23, 26 and 30 in N. oceanica. Blue and dark-red represented compartment A and B, respectively. Figure S8. Gene density and GC content in the transition region of compartment A/B under C0 vs. N1 and C0 vs. N2. A GC content in the compartment A/B switching region under C0 vs. N1 and C0 vs. N2. B Gene density in the compartment A/B switching region under C0 vs. N1 and C0 vs. N [file 13068_2023_2378_MOESM1_ESM.pdf]

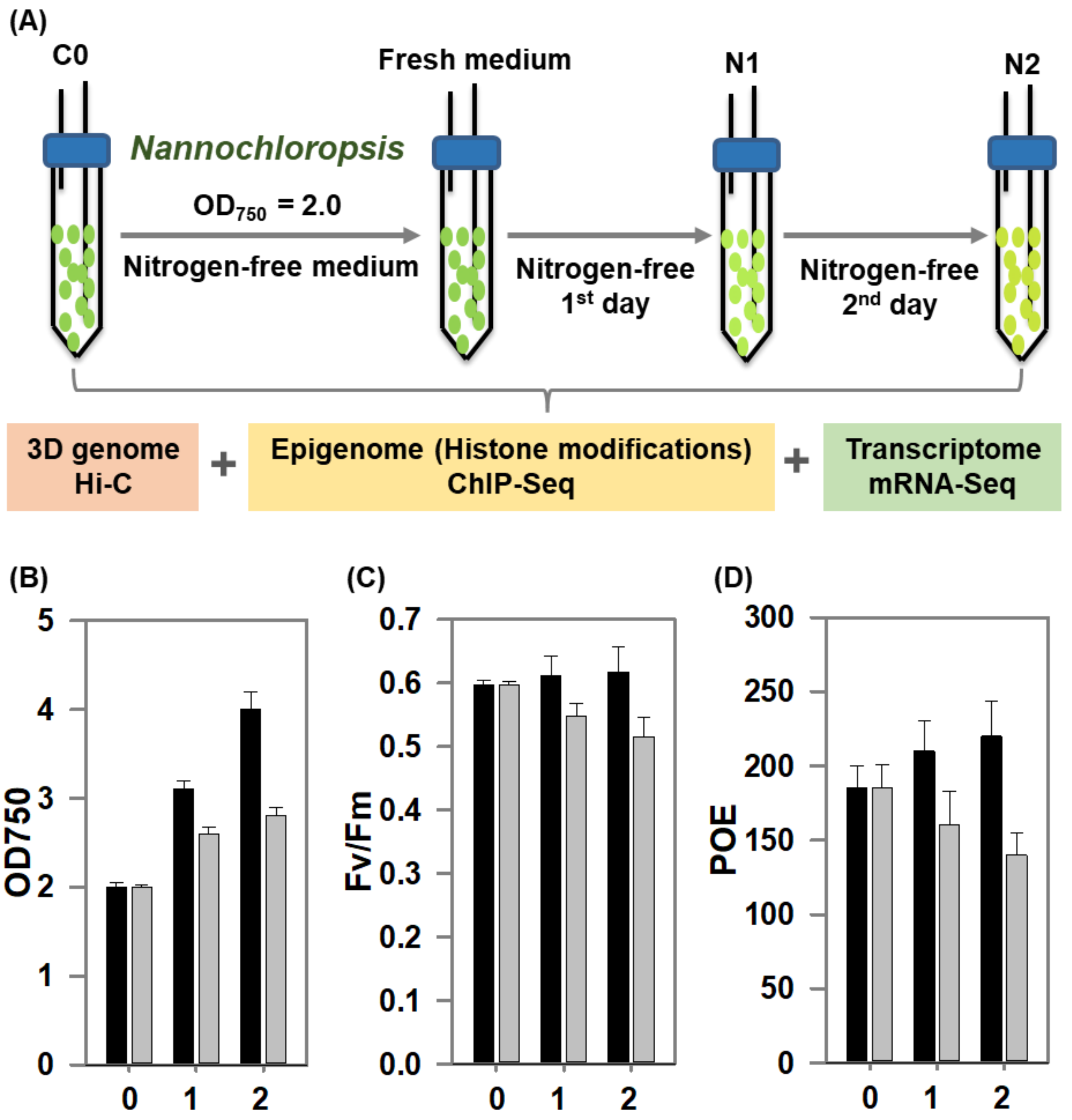

Figure S1. Experimental design and physiological measurement under N repletion and deprivation. (**A**) Experimental design for Hi-C, ChIP-seq and mRNA seq data collection. (**B**) OD750, photosynthetic parameter (Fv/Fm) and photosynthetic oxygen evolution (POE) under nitrogen repletion and deprivation.

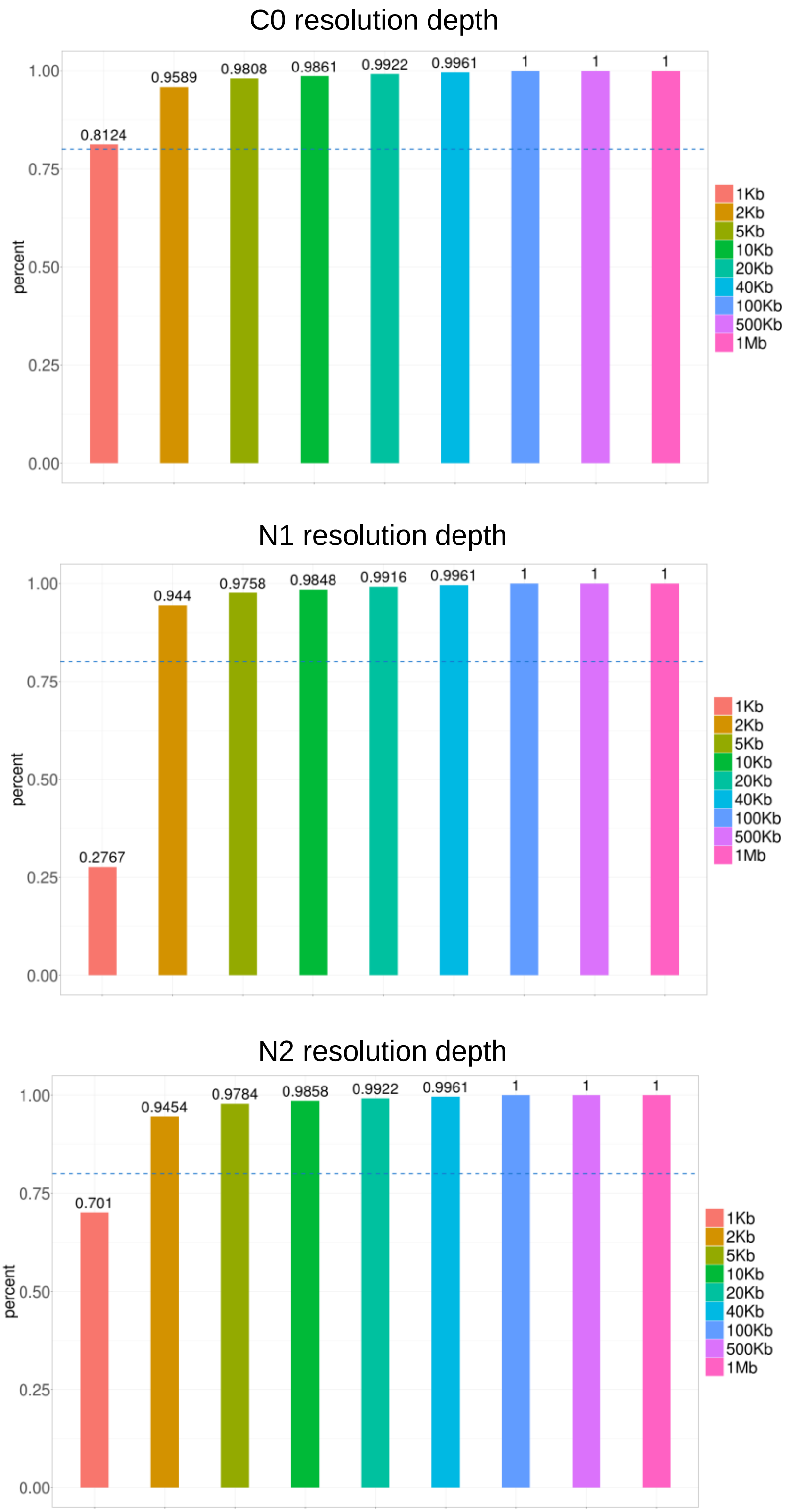

Figure S2. Depth in different resolutions. The matrix resolution of a Hi-C map was defined as the smallest locus size such that 80% of loci have at least 1,000 contacts. The resolution of >2kb is suitable for following analysis.

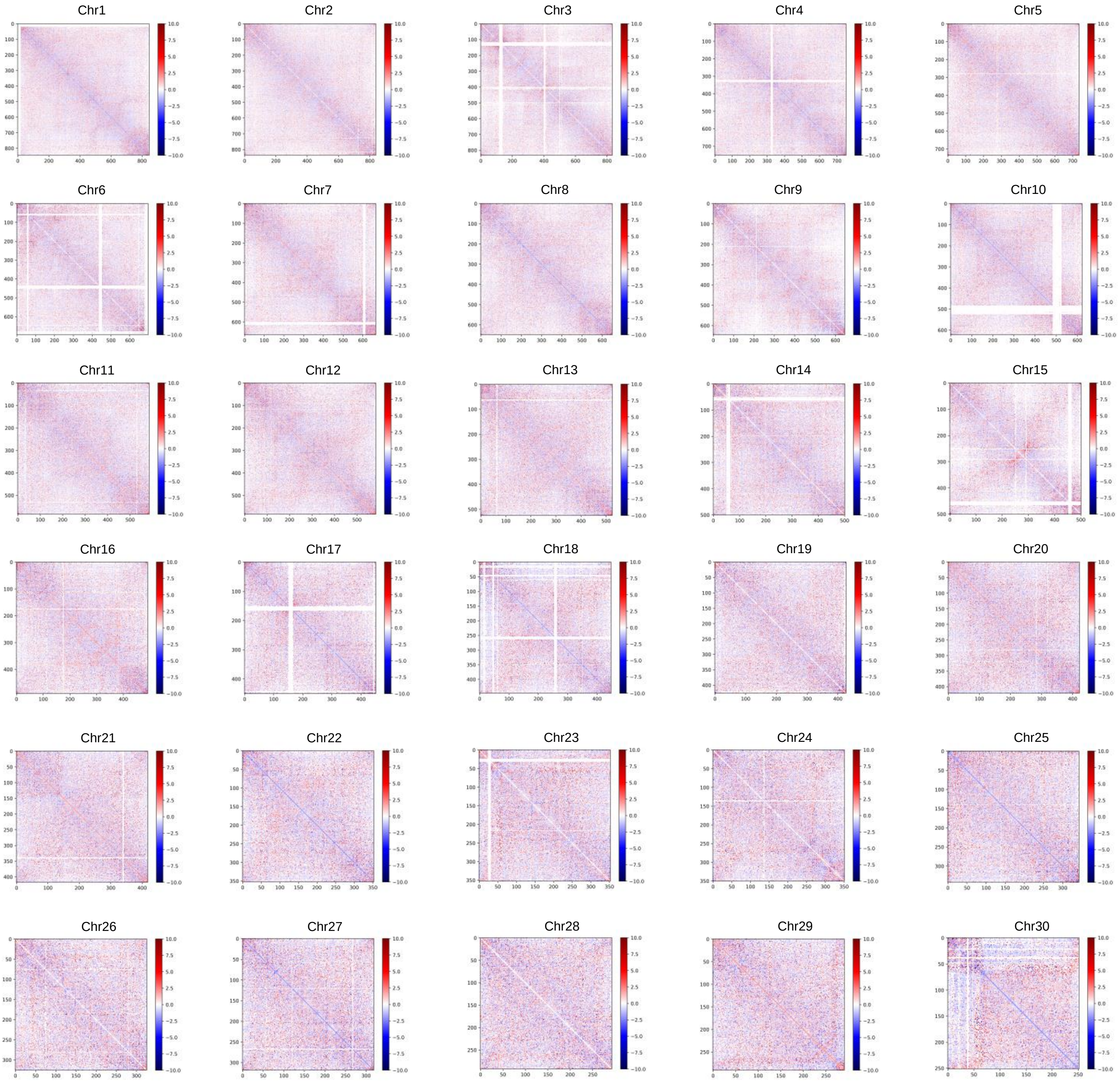

Figure S3. Minus heatmap matrix of each chromosome in response to nitrogen deprivation between C0 and N1. At 2kb resolution, substraction heatmap matrix of each chromosome was constructed between C0 and N1.

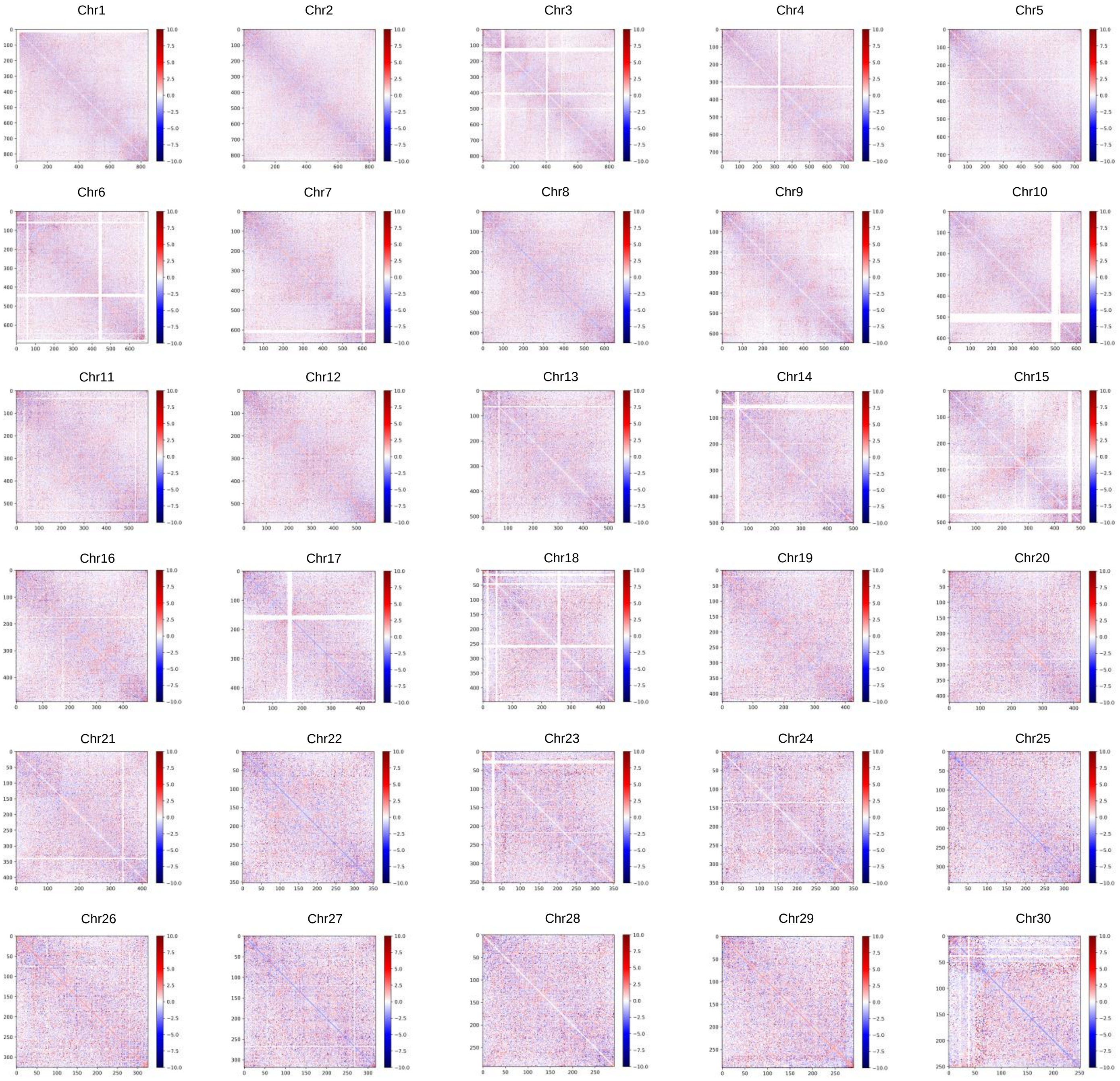

Figure S4. Minus heatmap matrix of each chromsome in response to nitrogen deprivation between C0 and N2. At 2kb resolution, substraction heatmap matrix of each chromosome was constructed between C0 and N2.

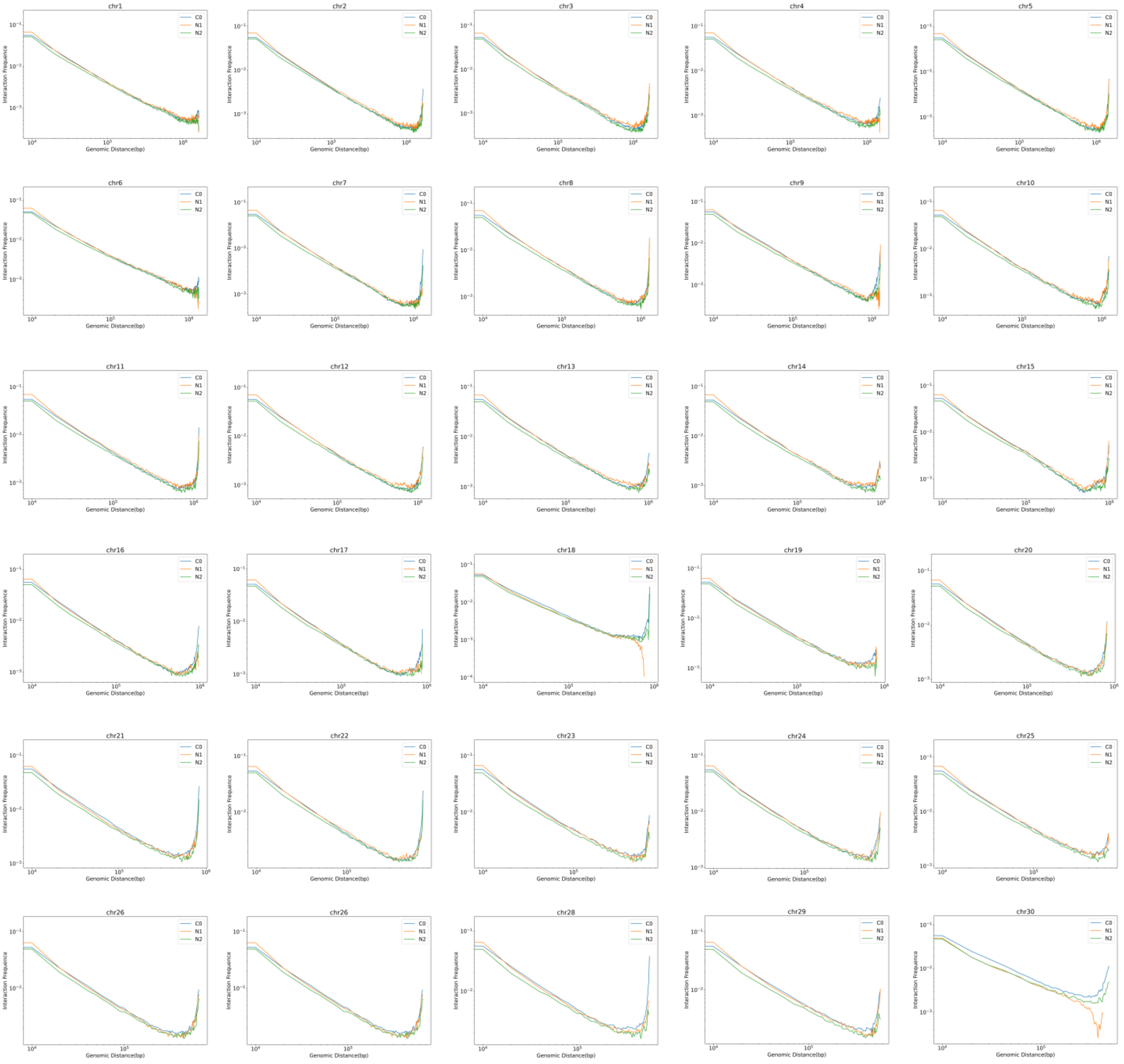

Figure S5. Dekay curve of interaction frequencies against increasing genomic distance for each chrosome in *N. oceanica*. The genomic bin size is 100kb.

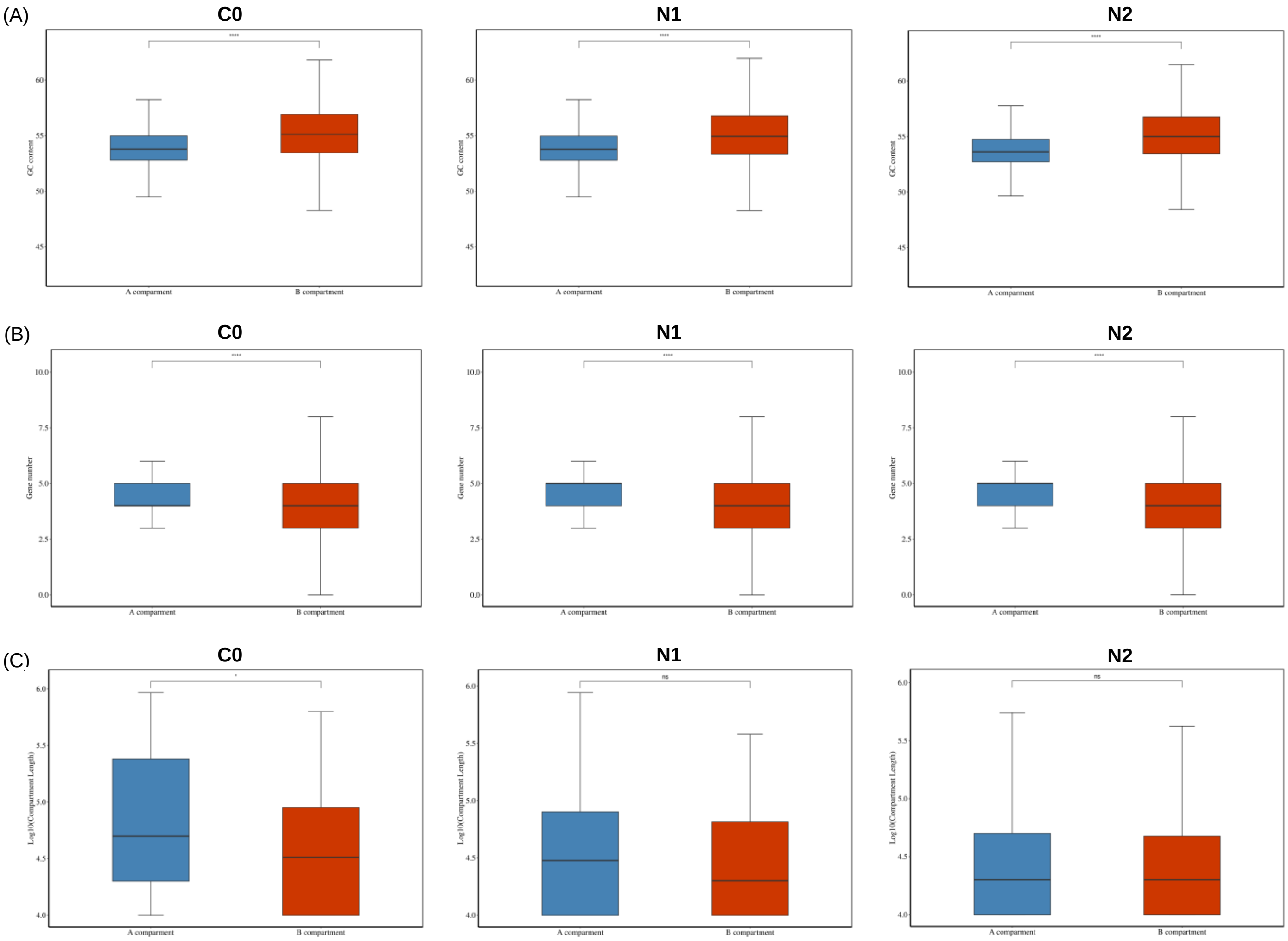

Figure S6. Gene density and GC content in the compartment A/B under C0 vs. N1 and C0 vs. N2. (**A**) GC content in the region of compartment A and B under C0, N1 and N2. (**B**) Gene density in the region of compartment A and B under C0, N1 and N2. (**C**) Comparison of compartment length in the region of compartment A and B under C0, N1 and N2.

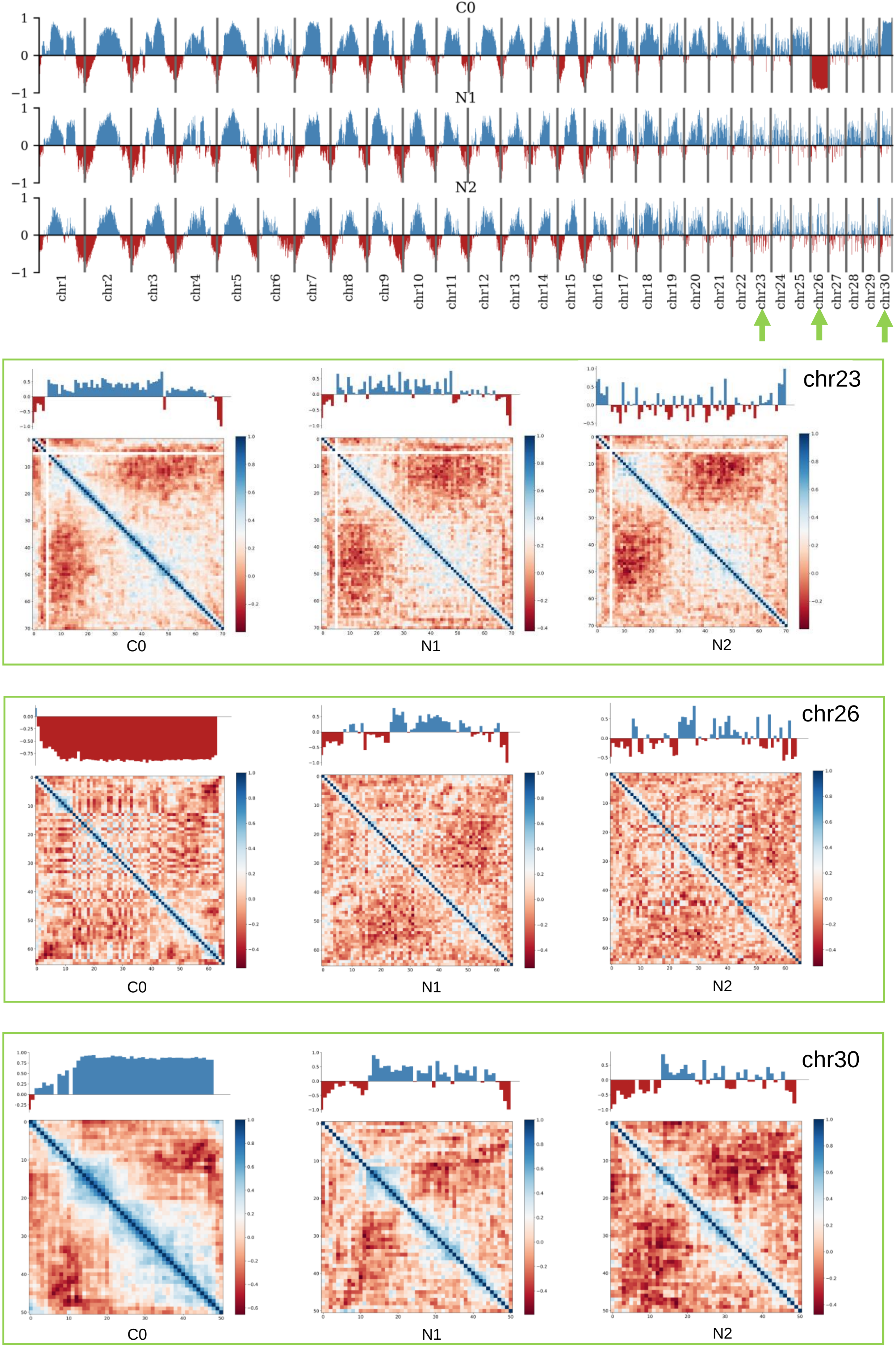

Figure S7. Genome eigenvector analysis of compartment A/B in chrosome 23, 26 and 30. Segregation of local A/B compartments using eigenvector under C0, N1 and N2 for chrosome 23, 26 and 30 in *N. oceanica*. Blue and dark-red represented compartment A and B, respectively.

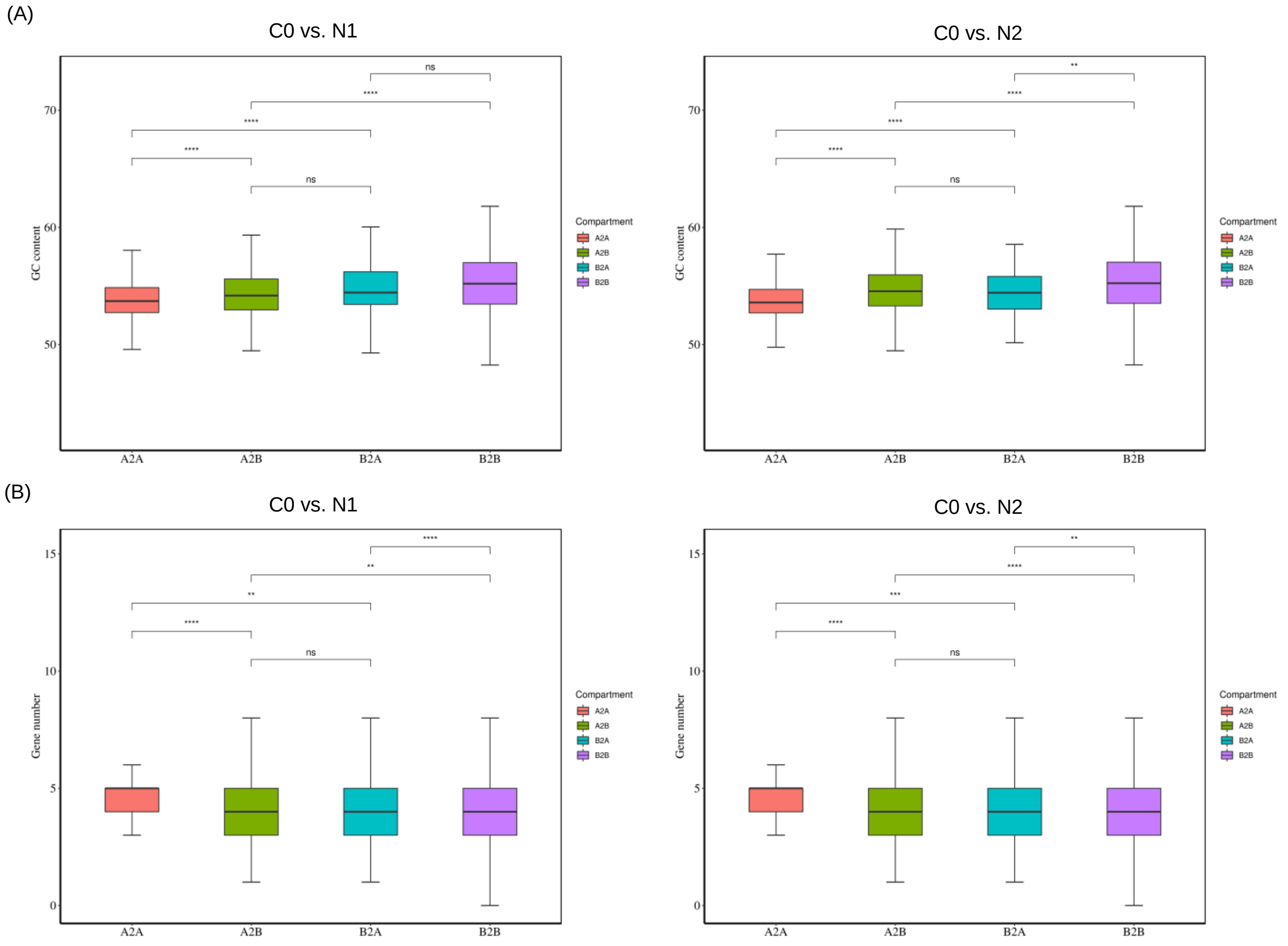

Figure S8. Gene density and GC content in the transition region of compartment A/B under C0 vs. N1 and C0 vs. N2. (**A**) GC content in the compartment A/B switching region under C0 vs. N1 and C0 vs. N2. (**B**) Gene density in the compartment A/B switching region under C0 vs. N1 and C0 vs. N2.

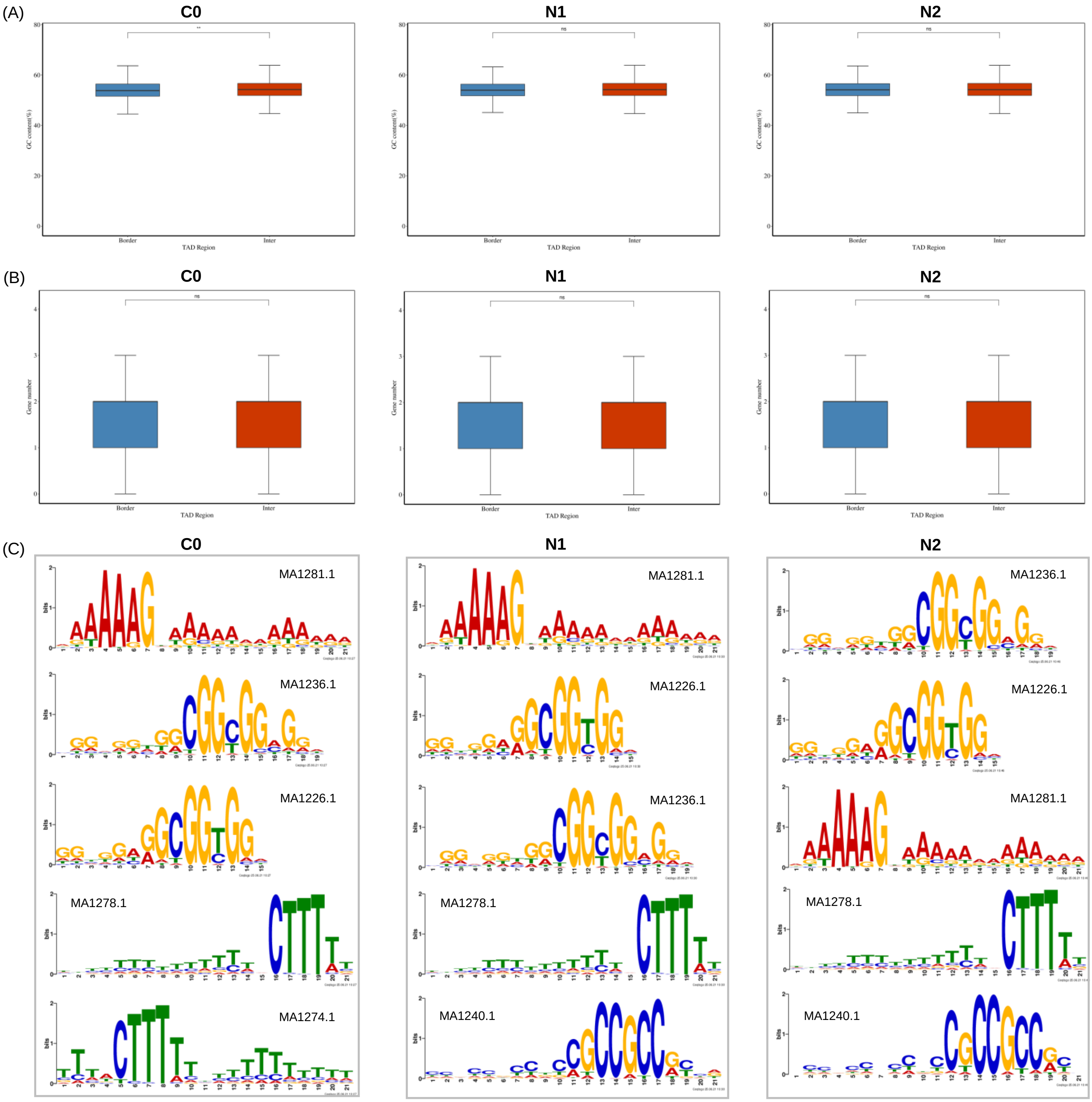

Figure S9. Gene density and GC content in the region of TAD border and inter under C0 vs. N1 and C0 vs. N2. (**A**) GC content in the region of TAD border and inter under C0, N1 and N2. (**B**) Gene density in the the region of TAD border and inter under C0, N1 and N2. (**C**) The top five motifs in the region of TAD border C0, N1 and N2.

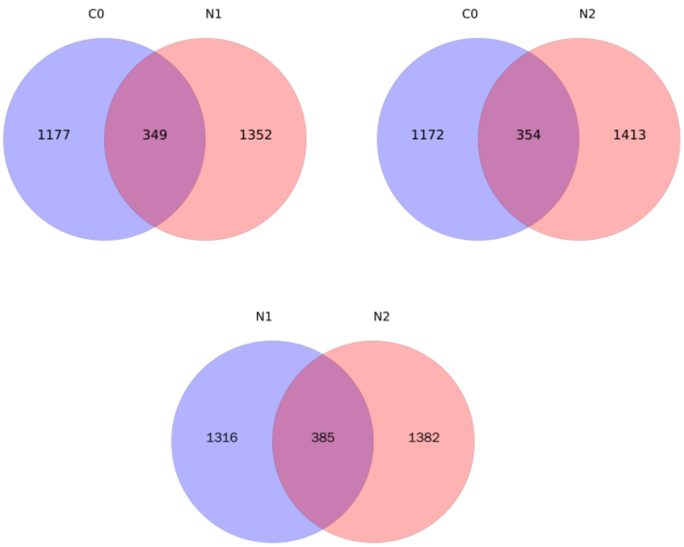

Figure S10. Venn diagram of TAD border between C0 and N1, or C0 and N2, or N1 and N2. The number of TAD border were compared between C0 and N1, or C0 and N2, or N1 and N2.

(A)

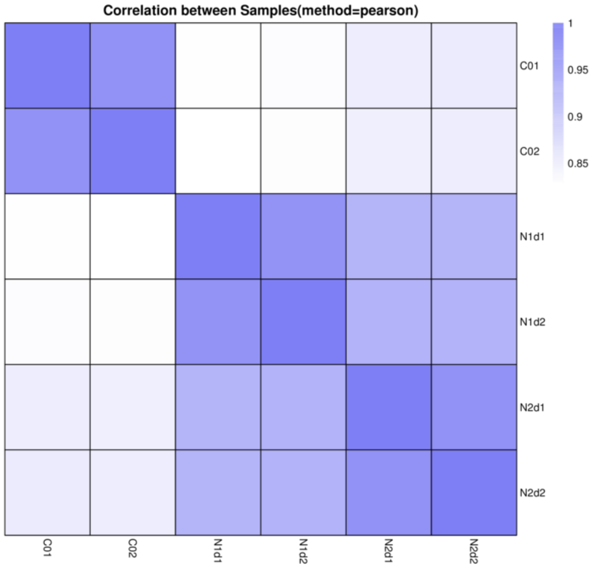

(B)

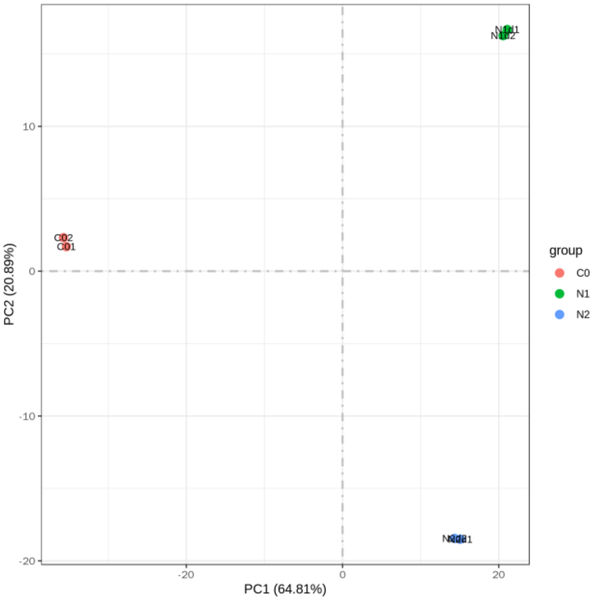

Figure S11. Pearson correlation relationships of mRNA-Seq libraries. (**A**) Pearson correlation relationships of six mRNA-Seq libraries. (**B**) PCA analysis of six sequencing samples.

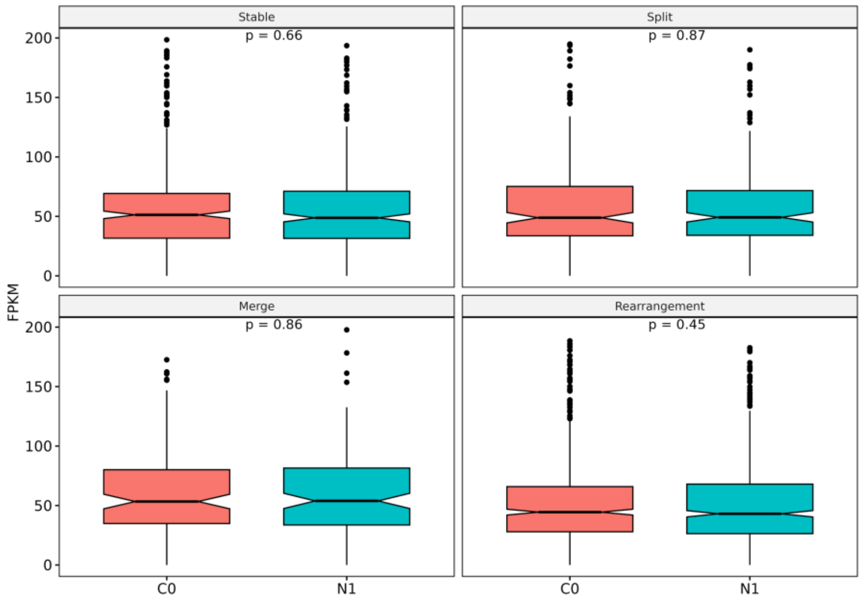

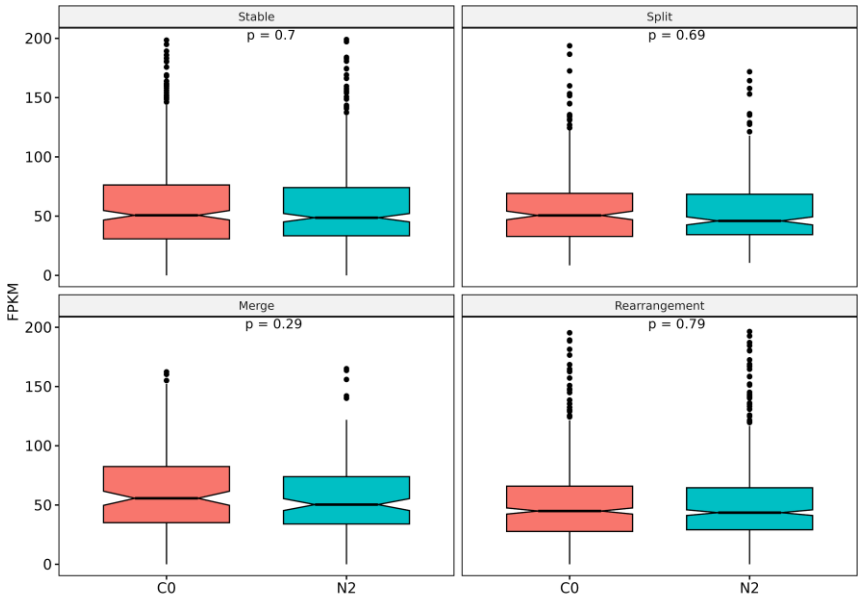

Figure S12. Comparsion of the gene expression in TAD transition regions under C0 vs. N1 and C0 vs. N2. (**A**) and (**B**) showed gene expression level in TAD transition regions under C0 vs. N1 and C0 vs. N2, respectively.

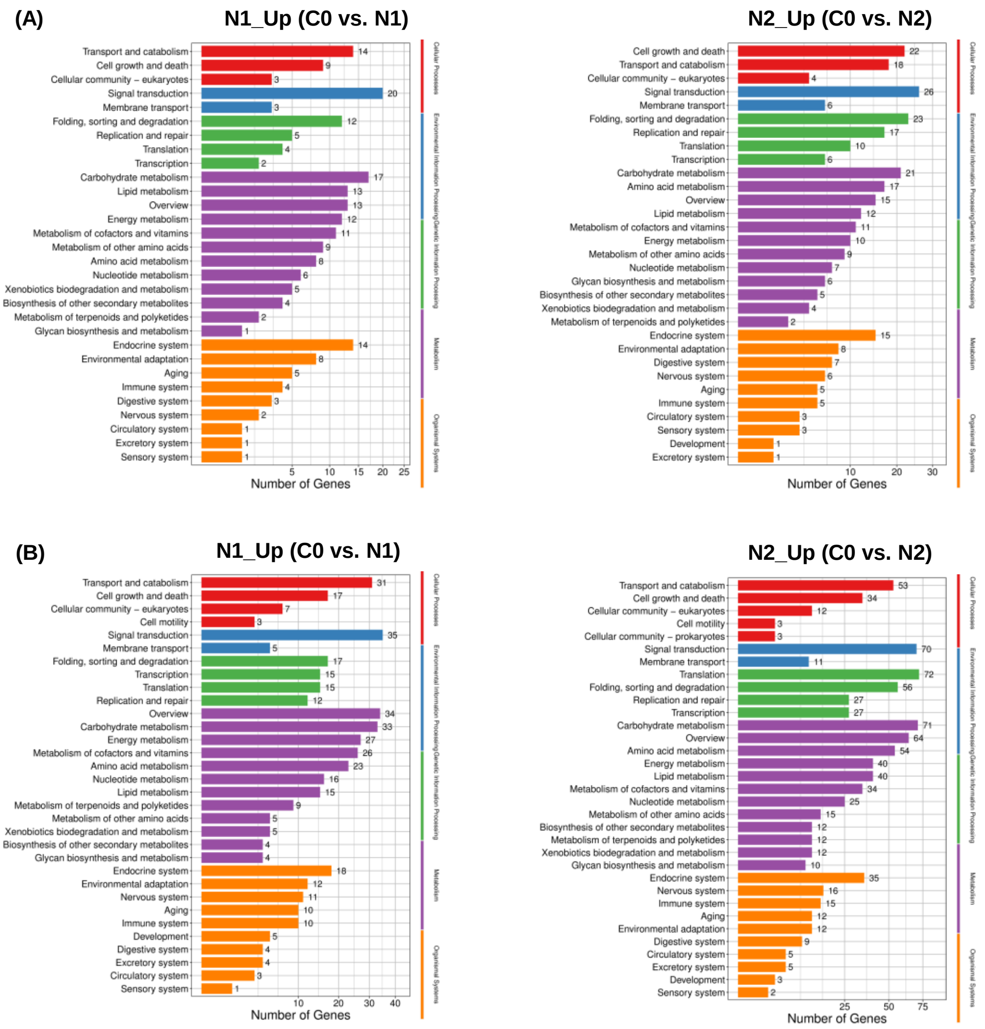

Figure S13. KEGG functional enrichment of up-regulated genes in compartment A/B and TAD under C0 vs. N1 and C0 vs. N2. (**A**) and (**B**) KEGG functional enrichment of up-regulated genes in compartment A/B under C0 vs. N1 and C0 vs. N2. (**C**) and (**D**) KEGG functional enrichment of up-regulated genes in the TAD bonder under C0 vs. N1 and C0 vs. N2.

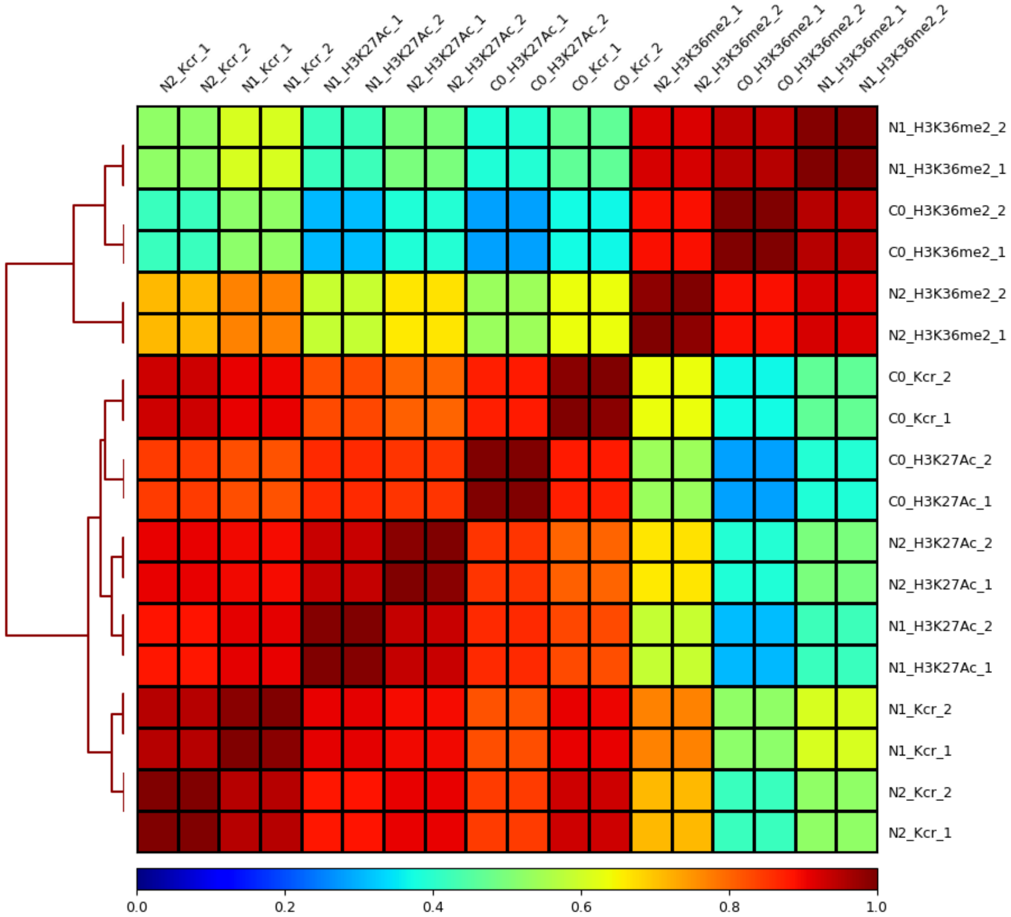

Figure S14. Correlation coefficients of ChIP-seq libraries. Heatmap showed correlation of different samples.

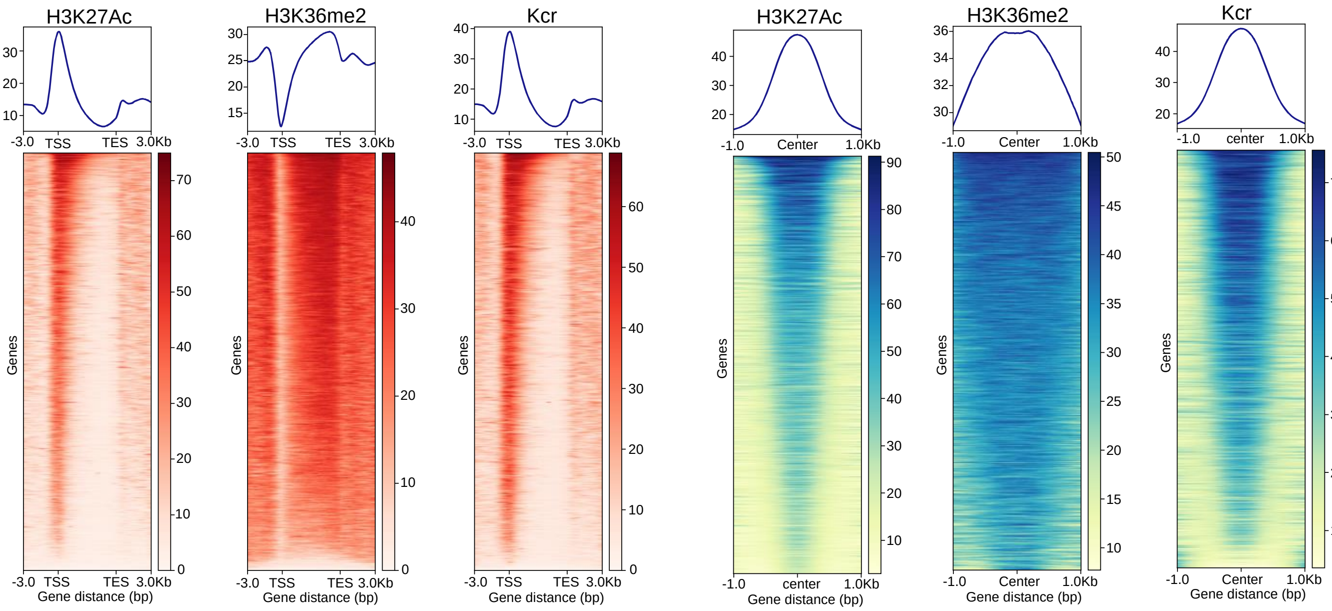

Figure S15. Distribution of enrichment analysis in histone modifications. Histone modification enrichment profile across genes were showed.

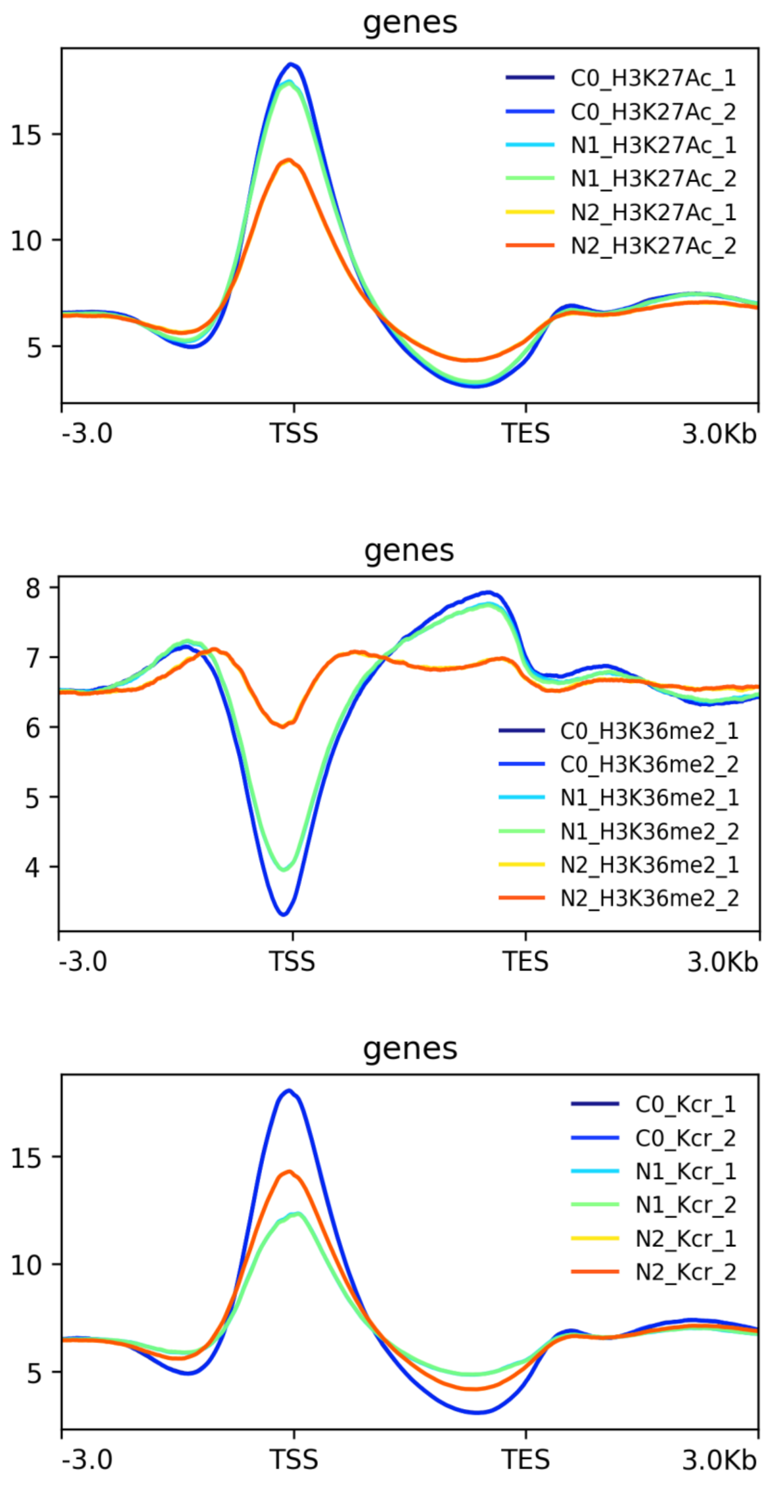

Figure S16. Pattern of histone modifications over 6kb regions surrounding the TSS for H3K27ac and H3K36me2.
