# Supplementary material for: Remodeling of the 3D chromatin architecture in the marine microalga Nannochloropsis oceanica during lipid accumulation
Source: Biotechnol Biofuels Bioprod. 2023 Aug 17;16:129. doi: 10.1186/s13068-023-02378-0 (PMC10436460; doi:10.1186/s13068-023-02378-0)
Supplement: Supplementary file 2 — Additional file 2: Table S1. Statistics of Hi-C sequencing data. Table S2. Percent of the genomic regions in compartment A/B. Table S3. Statistics of stable and switch compartment A/B. Table S4. Statistics of mRNA sequencing data. Table S5. Statistics of ChIP sequencing data. Table S6. Static of total peaks in three modifications (H3K27ac, H3K36me2 and Kcr). Table S7. Statistics of overlap peaks for H3K27ac, H3K36me2 and Kcr between C0 and N1, or C0 and N2. [file 13068_2023_2378_MOESM2_ESM.docx]

**Additional tables**

**Table S1.** **Statistics of Hi-C sequencing data.**

| **Samples** | **C0_1** | **C0_2** | **N1_1** | **N1_2** | **N2_1** | **N2_2** |
| --- | --- | --- | --- | --- | --- | --- |
| Sequencing data (kb) | 30,616,690 | 27,678,538 | 31,131,474 | 28,154,834 | 30,290,136 | 35,690,748 |
| Clean reads | 39,976,812 | 36,110,269 | 40,100,357 | 36,251,918 | 38,112,587 | 44,821,100 |
| Filter reads | 6,811,512 | 7,232,524 | 7,159,211 | 7,348,503 | 7,294,767 | 6,680,710 |
| Mapped reads | 33,165,300 | 28,877,745 | 32,941,146 | 28,903,415 | 30,817,820 | 38,140,390 |
| Duplicated reads | 2,445,997 | 2,239,650 | 939,861 | 777,343 | 1,519,483 | 1,720,992 |
| Valid pairs | 18,800,633 | 16,114,064 | 7,320,838 | 5,810,707 | 11,074,127 | 13,426,118 |
| Cis valid pairs | 8,530,242 | 7,326,206 | 3,907,476 | 3,128,182 | 5,175,436 | 6,259,433 |
| Trans valid pairs | 10,270,391 | 8,787,858 | 3,413,362 | 2,682,525 | 5,898,691 | 7,166,685 |

**Table S2.** **Percent of the genomic regions in compartment A/B.**

| **Sample** | **Compartment A (%)** | **Compartment B (%)** |
| --- | --- | --- |
| C0 | 63.11% | 35.13% |
| N1 | 57.70% | 40.63% |
| N2 | 53.20% | 45.02% |

**Table S3.** **Statistics of stable and switch compartment A/B.**

|  | **A-A** | **B-B** | **A-B** | **B-A** |
| --- | --- | --- | --- | --- |
| C0 vs. N1 | 1671 | 978 | 285 | 118 |
| C0 vs. N2 | 1553 | 997 | 403 | 97 |
| N1 vs. N2 | 1498 | 1112 | 290 | 152 |

**Table S4. Statistics of mRNA sequencing data.**

| **Samples** | **C0_1** | **C0_2** | **N1_1** | **N1_2** | **N2_1** | **N2_2** |
| --- | --- | --- | --- | --- | --- | --- |
| Clean Reads Pairs | 6,853,255 | 6.984.031 | 7,462,813 | 6,993,216 | 9,053,884 | 8,760,637 |
| Clean Base (bp) | 2,069,683,010 | 2,109,177,362 | 2,253,769,526 | 2,111,951,232 | 2,734,272,968 | 2,645,712,374 |
| Clean Q20  Bases Rate (%) | 97.3 | 97.4 | 97.3 | 97.0 | 95.7 | 95.7 |
| Clean Q30 Bases Rate (%) | 93.1 | 93.2 | 93.1 | 92.3 | 90.8 | 90.5 |

**Table S5. Statistics of ChIP sequencing data.**

| Sample | Raw Read Pairs | Sequencing data (Gb) | Clean Read Pairs | Proper pairs | Valid pairs |
| --- | --- | --- | --- | --- | --- |
| C0I1 | 8,645,041 | 2.53 | 8,445,036 | 3,640,655 | 2,629,406 |
| C0I2 | 8,945,063 | 2.71 | 8,565,142 | 3,741,652 | 2,789,515 |
| C0_H3K27Ac_1 | 10,688,875 | 3.21 | 10,688,870 | 5,965,458 | 4,551,476 |
| C0_H3K27Ac_2 | 11,517,594 | 3.46 | 11,517,590 | 6,577,695 | 4,973,663 |
| C0_H3K36me2_1 | 9,896,720 | 2.97 | 9,896,709 | 6,896,026 | 5,260,219 |
| C0_H3K36me2_2 | 9,996,728 | 2.98 | 9,996,710 | 6,995,015 | 5,365,216 |
| C0_Kcr_1 | 8,651,300 | 2.60 | 8,651,297 | 2,859,253 | 1,949,917 |
| C0_Kcr_2 | 9,593,841 | 2.88 | 9,593,838 | 3,255,189 | 2,172,472 |
| N1P1 | 9,203,165 | 2.76 | 9,203,153 | 5,097,626 | 3,970,193 |
| N1P2 | 9,303,112 | 2.82 | 9,301,052 | 5,187,535 | 3,996,182 |
| N1_H3K27Ac_1 | 5,347,046 | 1.60 | 5,347,016 | 2,334,507 | 1,857,058 |
| N1_H3K27Ac_2 | 6,150,319 | 1.85 | 6,150,313 | 2,631,718 | 2,070,665 |
| N1_H3K36me2_1 | 17,548,471 | 5.26 | 17,548,394 | 9,063,745 | 7,294,859 |
| N1_H3K36me2_2 | 16,592,884 | 4.98 | 16,592,868 | 8,352,849 | 6,687,899 |
| N1_Kcr_1 | 9,212,125 | 2.76 | 9,212,086 | 4,658,551 | 3,717,281 |
| N1_Kcr_2 | 8,210,165 | 2.46 | 8,210,156 | 4,071,416 | 3,229,736 |
| N2I1 | 9,652,281 | 2.90 | 9,652,274 | 5,412,030 | 4,008,958 |
| N2I2 | 9,542,265 | 2.86 | 9,542,245 | 5,352,081 | 4,218,681 |
| N2_H3K27Ac_1 | 9,047,852 | 2.71 | 9,047,849 | 2,629,304 | 1,957,807 |
| N2_H3K27Ac_2 | 10,102,584 | 3.03 | 10,102,583 | 3,019,662 | 2,223,978 |
| N2_H3K36me2_1 | 8,891,233 | 2.67 | 8,891,225 | 4,699,901 | 3,585,888 |
| N2_H3K36me2_2 | 10,753,221 | 3.23 | 10,753,211 | 5,862,650 | 4,396,932 |
| N2_Kcr_1 | 10,930,853 | 3.28 | 10,930,841 | 7,029,623 | 5,372,063 |
| N2_Kcr_2 | 8,257,929 | 2.48 | 8,257,923 | 6,011,512 | 4,579,352 |

**Table S6. Static of total peaks in three modifications (H3K27ac, H3K36me2 and Kcr).**

|  | **C0** | **N1** | **N2** |
| --- | --- | --- | --- |
| H3K27ac | 4,416 | 3,737 | 3,861 |
| H3K36me2 | 4,071 | 3,176 | 2,579 |
| Kcr | 4,327 | 4,315 | 3,980 |

**Table S7. Static of overlap peaks for ChIP-seq under C0 vs. N1 and C0 vs. N2.**

|  | **C0 vs. N1** | **C0 vs. N2** |
| --- | --- | --- |
| H3K27ac | 4,025 | 3,950 |
| H3K36me2 | 3,653 | 2,748 |
| Kcr | 3,885 | 4,245 |
